# Genome-wide characterization of microRNA in foxtail millet (*Setaria italica*)

**DOI:** 10.1186/1471-2229-13-212

**Published:** 2013-12-13

**Authors:** Fei Yi, Shaojun Xie, Yuwei Liu, Xin Qi, Jingjuan Yu

**Affiliations:** 1 State Key Laboratory of Agrobiotechnology, College of Biological Sciences, China Agricultural University, Beijing 100193, China

**Keywords:** miRNA, Foxtail millet, Expression pattern, Mirtron, miRNA*, Targets, Synteny

## Abstract

**Background:**

MicroRNAs (miRNAs) are a class of short non-coding, endogenous RNAs that play key roles in many biological processes in both animals and plants. Although many miRNAs have been identified in a large number of organisms, the miRNAs in foxtail millet (*Setaria italica*) have, until now, been poorly understood.

**Results:**

In this study, two replicate small RNA libraries from foxtail millet shoots were sequenced, and 40 million reads representing over 10 million unique sequences were generated. We identified 43 known miRNAs, 172 novel miRNAs and 2 mirtron precursor candidates in foxtail millet. Some miRNA*s of the known and novel miRNAs were detected as well. Further, eight novel miRNAs were validated by stem-loop RT-PCR. Potential targets of the foxtail millet miRNAs were predicted based on our strict criteria. Of the predicted target genes, 79% (351) had functional annotations in InterPro and GO analyses, indicating the targets of the miRNAs were involved in a wide range of regulatory functions and some specific biological processes. A total of 69 pairs of syntenic miRNA precursors that were conserved between foxtail millet and sorghum were found. Additionally, stem-loop RT-PCR was conducted to confirm the tissue-specific expression of some miRNAs in the four tissues identified by deep-sequencing.

**Conclusions:**

We predicted, for the first time, 215 miRNAs and 447 miRNA targets in foxtail millet at a genome-wide level. The precursors, expression levels, miRNA* sequences, target functions, conservation, and evolution of miRNAs we identified were investigated. Some of the novel foxtail millet miRNAs and miRNA targets were validated experimentally.

## Background

MicroRNAs (miRNAs) are 20 ~ 22 nucleotide (nt) non-coding small RNAs (smRNAs). Most primary miRNAs (pri-miRNAs) are transcribed from miRNA genes by RNA polymerase II (RNApol II), while others are transcribed by RNA polymerase III (RNApol III) [1]. In plants, the nascent pri-miRNA transcripts are first processed into 60 to 500 nt pre-miRNAs by DICER-LIKE1 (DCL1) [2]. Then, the pre-miRNAs undergo a second cleavage in the nucleus, releasing an RNA duplex containing the mature miRNA and the miRNA* sequences. To maintain the correct size and/or to protect them from polyuridylation [2,3], the duplexes are methylated at the 3′ ends by HUA ENHANCER [4] and then transported into the cytoplasm by HASTY [5,6]. The mature miRNA is incorporated into an RNA-induced silencing complex (RISC) that contains the ARGONAUTE (AGO) protein. The complex recognizes partially complementary sequences in the target messenger RNA (mRNA) where it binds, inducing its target mRNA’s degradation or a combination of both degradation and translation inhibition [7]. The miRNA* is released and rapidly degraded (had ~9% as many reads as the mature miRNAs) [8]. The presence of the miRNA* has been regarded as a good standard way to reliably annotate a novel miRNA [9].

Mirtrons are a new, recently discovered, type of miRNAs that originate from spliced introns of gene transcripts. Mirtrons reside within the intronic regions of genes and are processed through a Drosha-independent pathway, making them quite distinct from the other miRNAs. The mirtron pathway was first identified in *Drosophila melanogaster*, *Caenorhabditis elegans*, and some mammals [10-12] and has recently been found in *Arabidopsis thaliana* and *Oryza sativa*[13,14]. Recent research shows that the miRNAs from the intronic regions of genes can survive together with their host genes and support their host genes by mediating synergistic and antagonistic regulatory effects [15,16].

In plant, miRNAs are known to play crucial roles in many developmental events [17] and regulate target transcripts through two modes of action: degradation and translation inhibition [18,19]. The miRNA degradation occurs through miRNA-guided transcript cleavage in plants [20]. Although the mechanisms involved in the translational inhibition by miRNAs are largely unknown, recent studies from Xuemei Chen’s group show that the miRNA translational inhibition occurs at the endoplasmic reticulum (ER) and that ALTERED MERISTEM PROGRAM1 (AMP1) activity allows for the dissection of miRNA-mediated target RNA cleavage from miRNA-mediated target translation inhibition [7].

With the advent of second generation sequencing technology, the rate of miRNA discovery has increased dramatically [21-25]. In dicotyledonous species, 337 mature miRNAs in *A. thaliana*, 401 in *Populus trichocarpa*, and 164 in *Nicotiana tabacum* have been discovered, and, in monocotyledonous species, 713 mature miRNAs in *O. sativa*, 16 in *Saccharum offcinarum*, 241 in *Sorghum bicholor*, 42 in *Triticum aestivum* and 321 in *Zea may*s have been reported (miRBase; release 20, June 2013). Although foxtail millet is an important cereal crop, only a few miRNAs in foxtail millet have been reported. Bennetzen *et al*. [26] reported some known miRNAs from the alignment of mature miRNA sequences, but they did not investigate whether or not the corresponding precursors existed in the genome.

Foxtail millet is a diploid C4 panicoid crop species [27]. It is an important grass crop (family Poaceae) that has been planted widely in China. The genome of foxtail millet (Yugu1) has been sequenced [26], making it a possible genetic resource that could be used to investigate plant architecture, genome evolution and physiology in the bioenergy grasses. Because of the important roles that miRNAs play in gene regulatory networks [17], it is important to identify the miRNAs in foxtail millet and to investigate their potential target genes to gain a better understanding of the biological processes in this plant. In the present study, we aimed to characterize the miRNA repertoire of foxtail millet.

## Results

### Analysis of smRNAs in foxtail millet

To identify miRNAs in foxtail millet, we sequenced smRNAs for shoots (14-day-old) by high throughput Illumina sequencing technology [28]. Nearly 40 million reads of 18 ~ 31 nt, representing over 10 million unique sequences, were generated. In addition, three smRNA datasets (leaf, flower and root) of foxtail millet were downloaded from the Comparative Sequencing of Plant Small RNAs web site [29]. These datasets contained over 14 million smRNAs of 18 ~ 31 nt, representing over 4 million unique sequences. An analysis of the data from the four datasets (shoot, leaf, flower and root) revealed that a large number of sequences appeared only once. The percentages of these singletons were 84.03% (8,851,929) in shoot, 85.49% (792,474) in leaf, 83.43% (1,972,156) in flower and 82.53% (948,382) in root. In previous reports, only 65% and 82% singletons were found in *Arabidopsis*[8] and rice [22], respectively, suggesting that the smRNAs in foxtail millet are as complex as they are in rice, and more complex than in *Arabidopsis*.

**Figure 1:**
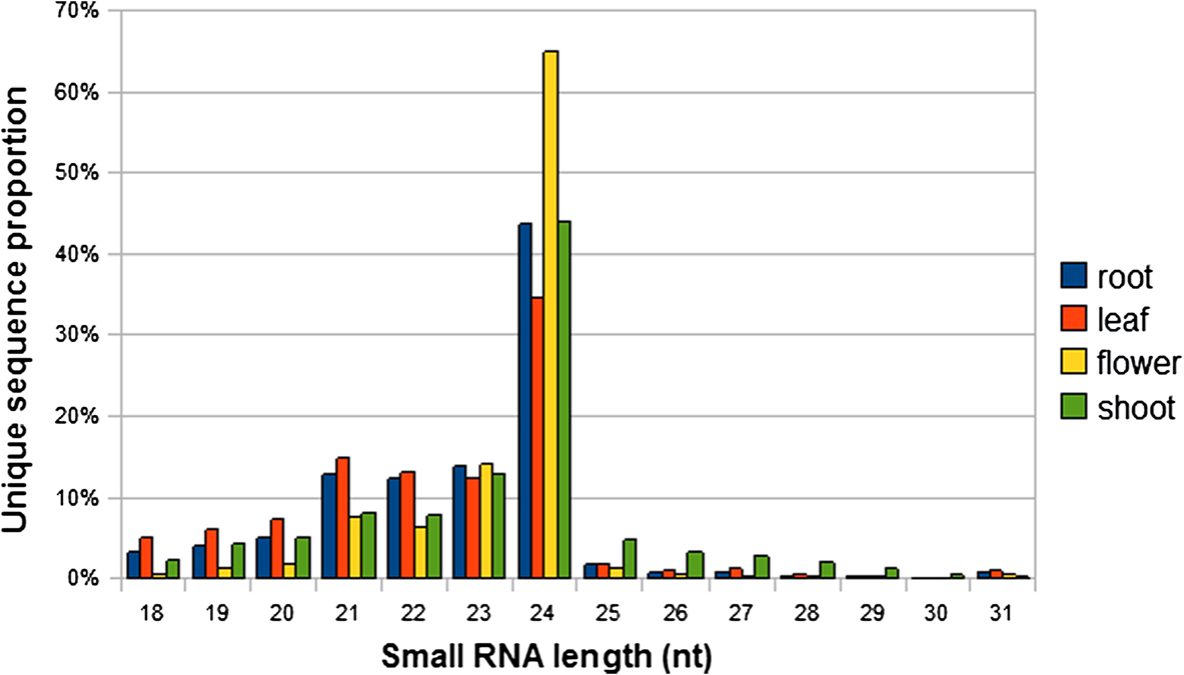
**Distribution of the lengths of the unique smRNA sequences.** Distribution of the sequence lengths of the smRNAs derived from root, leaf, flower and shoot generated by deep-sequencing. Counts were based on the unique sequences rather than the number of reads per unique sequence.

The overall size distribution of the unique reads from four sequencing efforts were very similar, with the 24 nt smRNAs being the most abundant, followed by the 23 nt and 21 nt smRNAs (Figure 1), which differ from the size distribution of total reads (Additional file 1A). A detailed comparison of the smRNAs derived from unique reads (Figure 1) or total reads (Additional file 1A) revealed some features of smRNA species. Frist, the 24 nt smRNAs dominated the pool of unique species in foxtail millet as observed for many other species such as *A. thaliana*[30] and cucumber [31]. Second, the 21 nt smRNAs replicated in even higher frequencies (ratio of total reads to unique reads) than 24 nt smRNAs in all four tissues. The average times (total reads/unique reads) of 21 nt are nearly 7.8, 9.6, 6.0 and 9.4 and the average times of 24 nt are nearly 2.1, 1.9, 1.9 and 2.0 in shoot, leaf, flower and root, respectively. Third, miRNAs, most of which were 20 to 22 nt in length, were relatively abundant in root and leaf, while siRNAs, most of which were 24 nt long, were relatively more prevalent in flower and shoot. These results were consistent with those reported previously in maize [32,33].

In foxtail millet, smRNA populations in four tissues are extremely complex. Although we have over 54 million reads, sequences of smRNAs have a limited overlap from the four databases (Additional file 1B). Only 79,225 unique sequences appeared in all four tissues and a small portion of the sequences overlapped between two tissues. The smRNAs in each tissue accounted for almost half of all the smRNAs in the combined datasets. This limited overlap indicated that there was a diversity of smRNAs in foxtail millet.

### Identification of known miRNAs in foxtail millet

In plants, miRNAs are obtained by the precise excision of ~21 nt smRNAs from the stem of a single-strand, stem-loop precursor. As a result, miRNAs can be identified by looking for a potential fold-back precursor structure that contains the ~21 nt miRNA sequence within one arm of the hairpin. The hairpin must have the lowest free energy of all the alternative folds for that sequence, as predicted by RNA folding programs such as Mfold [34] or RNAfold [21,32]. The minimal folding free energies index (MFEI) can be treated as the main feature to distinguish potential miRNA precursors from other RNAs. It has been reported that more than 90% of miRNA precursors have a MFEI value greater than 0.85, while the MFEI for other RNAs [tRNAs (0.64), rRNAs (0.59) and mRNAs (0.65)] are lower [35]. We used a previously reported workflow [21] to identify miRNAs among the smRNAs from foxtail millet, but with the MFEI values set to 0.85 rather than to 0.15 as was done previously. We identified 215 candidate miRNAs in the foxtail millet smRNA datasets; 137 in shoot, 64 in leaf, 62 in flower and 79 in root. The proportion of miRNA reads (those identified in the study) in each library as compared to the total reads were 3.0% in root, 4.1% in leaf, 1.4% in flower and 1.3% in shoot.

Among the 215 miRNAs, 43 unique mature miRNA sequences belonging to 19 miRNA families were 100% identical to mature miRNAs from 14 well-studied species [26]. We defined the 43 unique mature miRNAs in foxtail millet as known miRNAs (hereafter referred to as sit-miRNAs). There were 24 known miRNAs in root, 23 in leaf, 19 in flower and 35 in shoot tissues (Additional file 2). The 43 sit-miRNAs corresponded to 89 pre-miRNAs, of which 65 had corresponding miRNA* forms. Detailed information about the known sit-miRNAs is shown in Additional file 2.

**Figure 2:**
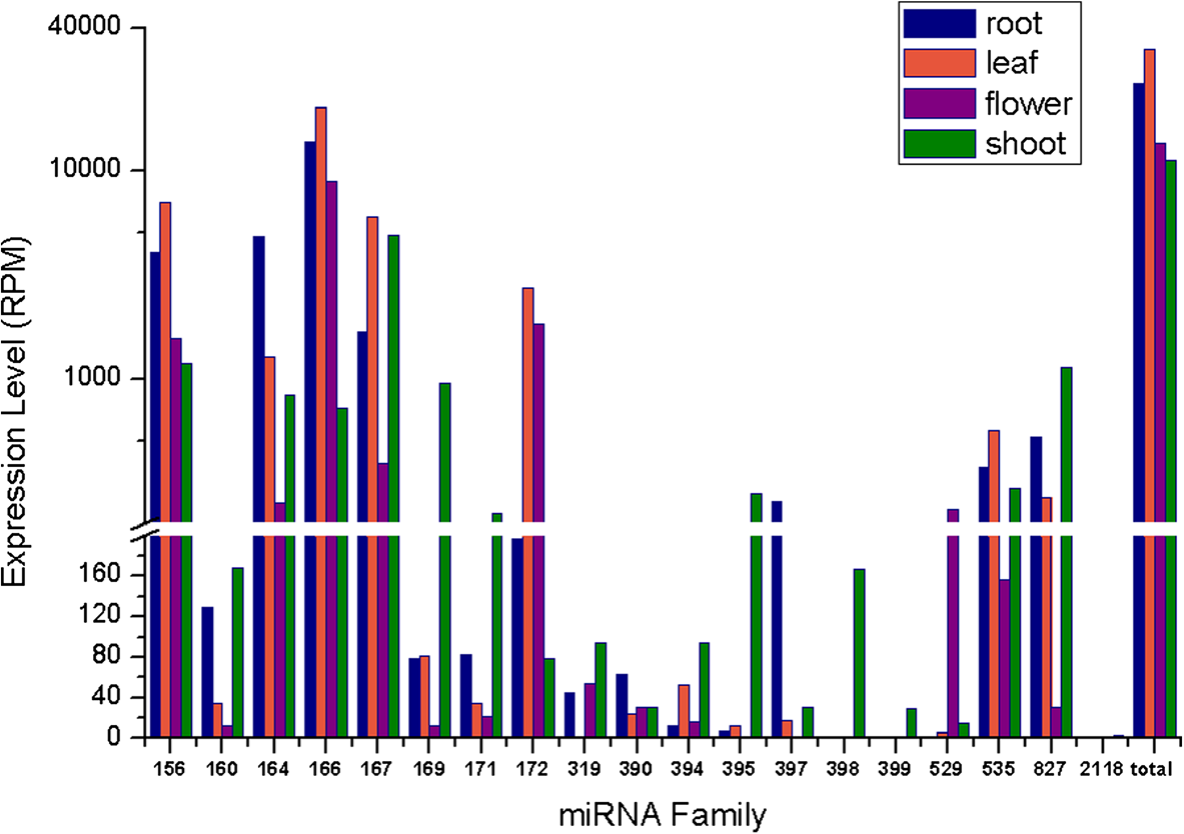
**Expression levels of 19 known miRNA families in four tissues from foxtail millet.** The expression levels of the miRNA families in each tissue were normalized by the total number of reads in each of the respective libraries. Counts were in reads per million (RPM).

A summary of the total expression profiles for each of the 19 miRNA families to which the 43 sit-miRNAs belong is shown in Figure 2. The number of miRNA reads were enumerated and normalized against the total count of smRNA reads, reported as reads per million (RPM), for each respective library. The results showed that the total expression of these 19 miRNA families was relatively higher in leaf and root than that in shoot and flower. We noted that the sit-miR156, sit-miR164, sit-miR166, sit-miR167 and sit-miR172 families showed relatively higher expression (slightly over 1,000 RPM, on average) in one or more of the four tissues. In contrast, the sit-miR319, sit-miR390, sit-miR394, sit-miR399 and sit-miR2118 families showed low expression (less than 100 RPM) levels. In our samples, the following miRNA families showed distinct expression patterns: miR529 is barely detected in roots; miR319 is practically absent in leaves; miR395 and miR397 are practically absent in flowers; miR398 and miR399 are detected only in shoots; and miR2118 is barely detectable in any of the four tissues.

**Figure 3:**
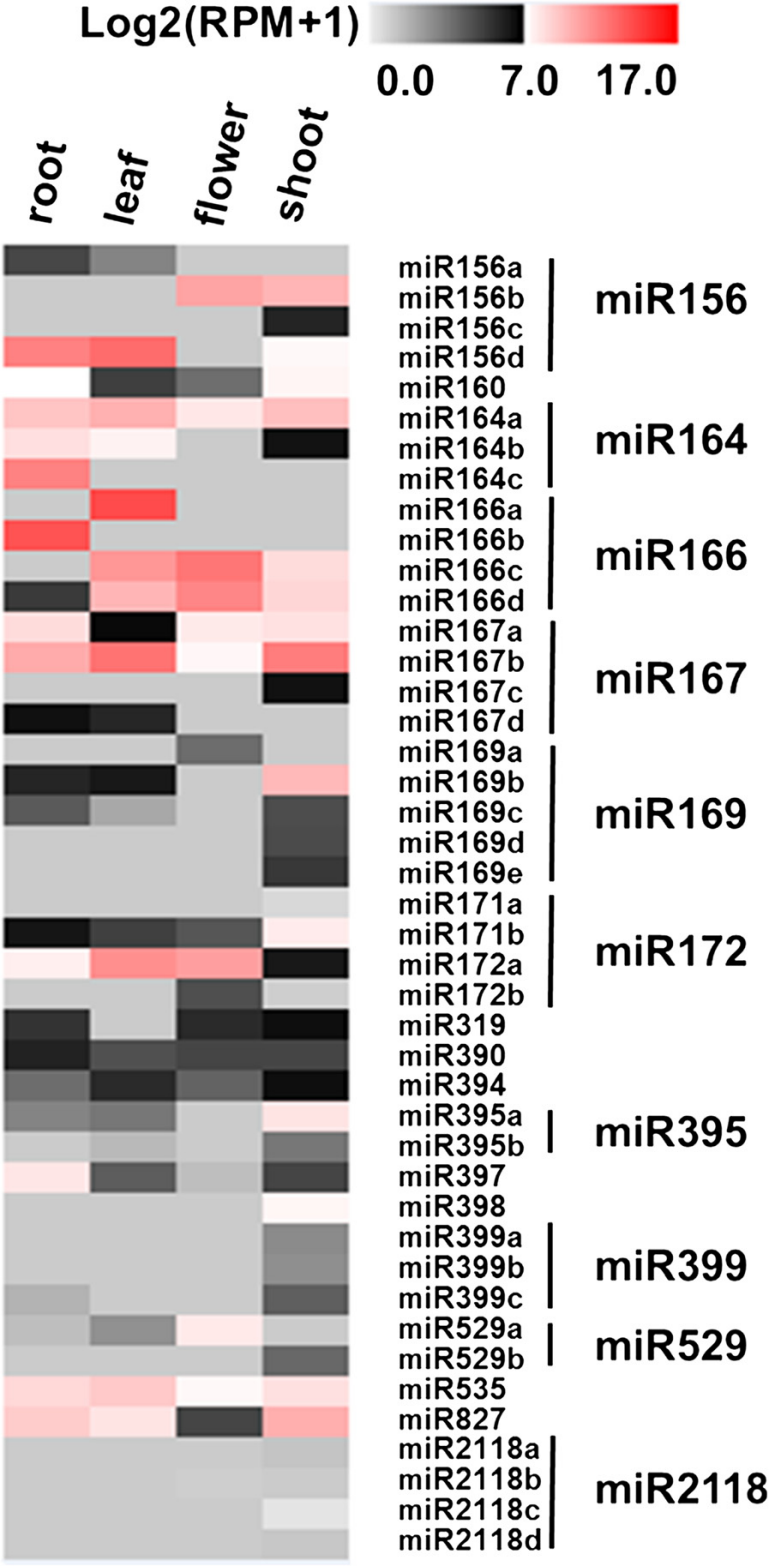
Heat map of expression profiles for all known miRNAs across four tissues in foxtail millet.

Our data also showed that some members of the same miRNA family had different tissue-specific expression patterns (Figure 3). For example, based on RPM counts, miR156b is highly expressed in flower and shoot but has almost no expression in leaf and root. While the miR156d is highly expressed in leaf and root but has almost no expression in the flower. This should be confirmed by other molecular techniques. Together, these results suggest that members from different families and different members from the same family may have greatly different effects on foxtail millet development.

### Novel miRNA identification

Our results showed that in addition to the 43 known mature sit-miRNAs, there were 172 novel miRNAs (hereafter referred to as nov-sit-miRNAs) in the smRNA datasets that have never been reported in other species. Because some of these miRNAs were derived from multiple precursors, these 172 nov-sit-miRNAs corresponded to 212 pre-miRNAs, 68% of which were located in intergenic regions. Only a few of the 212 pre-RNAs were located in introns or in the UTRs of coding genes, which is in agreement with the reported statistical characteristics of plant pre-miRNAs [3] (Additional file 3).

Among the 172 nov-sit-miRNAs, nine were found in all four libraries, nine were in three libraries, 25 were in two and 129 were in one library. These potentially nov-sit-miRNAs had relatively lower expression levels than the 43 known sit-miRNAs. For example, in leaf, the total expression levels of the 172 nov-sit-miRNAs (2267 RPM) were less than 1% of the total expression levels of the 43 sit-miRNAs (38438 RPM) and, in flower, the total expression levels of all the nov-sit-miRNAs were only 659 RPM. These results were consistent with a study in rice that found that most of the non-conserved miRNAs exhibited tissue-specific expression patterns and had relatively low expression levels compared with known miRNAs [22]. For example, nov-sit-miR43, nov-sit-miR125 and nov-sit-miR159 were expressed only in flowers with expression levels of more than 30 RPM, and nov-sit-miR104 and nov-sit-miR149 were expressed only in roots with expression levels of more than 180 RPM (Additional file 3).

**Figure 4:**
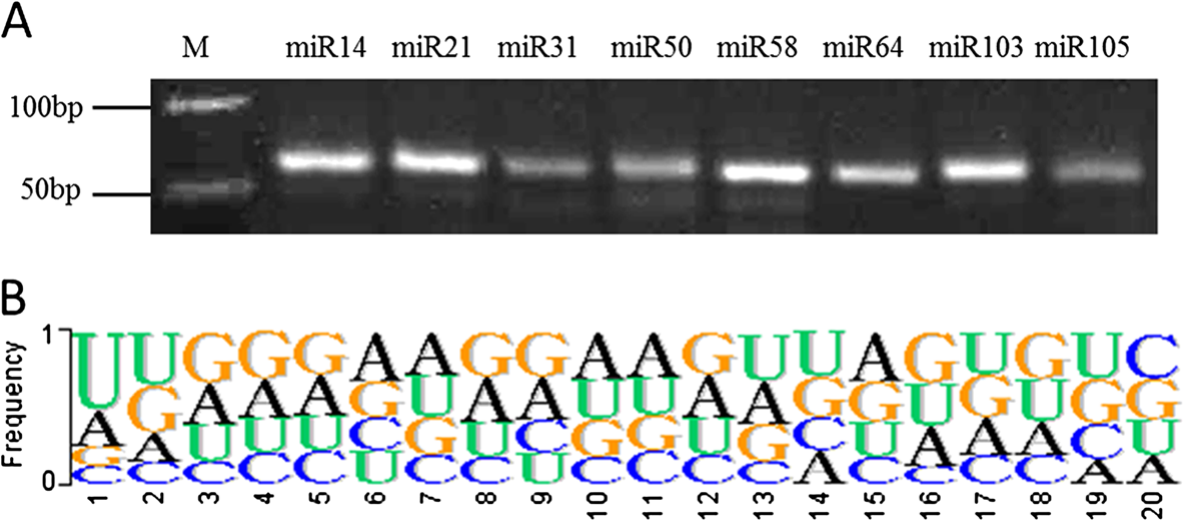
**Verification and characteristics of the novel foxtail millet miRNAs (nov-sit-miRNAs). (A)** The nov-sit-miRNAs were validated by stem-loop RT-PCR. The RNA used for stem-loop RT-PCR was isolated from shoots (14-day-old). **(B)** Nucleotide frequency of novel miRNAs.

The conservation of miRNA sequences across species could be regarded as powerful evidence to annotate miRNA [9]. We identified conserved miRNA by allowing no more than three mismatches between the mature miRNA sequences in our datasets and the mature miRNA sequences from 14 well-studied species [26] published in miRBase (release 20, June 2013). We identified 33 nov-sit-miRNAs that could be assigned to 14 published miRNA families based on sequence similarity (Additional file 3). These ‘conserved’ nov-sit-miRNAs generally had higher expression levels than the ‘non-conserved’ nov-sit-miRNAs. Eight of the nov-sit-miRNAs were validated experimentally by stem-loop RT-PCR and sequencing (Figure 4A). The presence of a miRNA* could be regarded as additional support for the annotation of the nov-sit-miRNAs [9]. We noted that 87 of the 212 novel pre-miRNAs were found having miRNA*s. A nucleotide composition analysis showed that 50% of the novel pre-miRNAs started with U (Figure 4B and Additional file 3), which is consistent with the statistical characteristic of mature miRNAs [36], and, overall, a higher percentage of A and U compared with C and G was present. Detailed information about novel miRNAs was shown in Additional file 3 and second structure of some novel miRNA precursors were presented in Additional file 4.

To predict mirtrons, we analyzed the secondary structure features of the introns of the foxtail millet genes that were annotated in the Phytozome database (see the Methods section for details). Introns can be folded directly to acquire stem–loop structures similar to the pre-miRNAs. Using RNAfold, we found many introns that had reasonable second structures; that is, a single stem-loop with less than 3 nt overhangs at both ends, and a minimum energy of -25 kcal/mol or less. After mapping the smRNAs in our datasets onto the selected introns using the BLAST algorithm (see the Methods section for detail), we found only a few perfectly matched smRNAs; therefore, only two mirtron precursor candidates could be identified (PAC: 19709968 and PAC: 19675165) (Additional file 5).

### Target prediction and expression validated

MiRNAs are known to have diverse expression patterns and play regulatory roles in various developmental and physiological processes [17]. In plants, most miRNAs are known to regulate development by mediating the destruction of their target mRNAs [37]. The target gene has sites that are almost completely complementary to the miRNA, so, in plants, miRNA target genes can be predicted computationally. To increase the reliability of our target prediction, we used the modified scoring described by Sun *et al*. [38]. We used the sit-miRNA and nov-sit-miRNA sequences in our datasets to search for complementary annotated foxtail millet transcript genes in the Phytozome database [39] and identified 447 candidate target genes for 166 of the foxtail millet miRNAs (Additional file 6).

We randomly picked six target genes to validate using 5′RACE and four were successfully validated (Si016508m, Si005991m, Si016509m and Si034525m) (Table 1). The Si016508m protein, which is encoded by one of the predicted target genes, is a GRAS (GAI, RGA, SCR) transcription factor that has been reported to be involved in development and other processes [40,41]. The Si016508m gene is potentially targeted by five of the sit-miRNAs (sit-miR171a, sit-miR171b, nov-sit-miR15, nov-sit-miR14 and nov-sit-miR49). The Si005991m, Si016509m and Si034525m protein, which are encoded by a predicted target gene (targeted by sit-miR160), is known to have three different domains: a B3 DNA-binding domain, an Aux/IAA-ARF dimerization motif, and a DNA-binding pseudobarrel domain. Previous studies have suggested that for efficient cleavage by miRNAs, base pairing between bases 10 and 11 is essential [42-45]. The 5′RACE results showed that the Si016508m gene had two breakpoints (at positions 1085 bp and 1082 bp). The predicted target sites of these genes showed that sit-miR171a and sit-miR171b guided Si016508m cleavage at 1085 bp, while nov-sit-miR14, nov-sit-miR15 and nov-sit-miR49 guided Si016508m cleavage at 1082 bp (Table 1). To show that these miRNAs, which have validated their targets, are actually expressed, we used the stem-loop RT-PCR method of detection. We detected miRNA160, miRNA171a, nov-sit-miR14, nov-sit-miR15 and nov-sit-miR49 successfully. However, we failed to detected miRNA171b (Additional file 7).

**Table 1:** Validated miRNA targets

**Target ID miRNA ID**	**Target start-end**	**5′-target sequence-3′ complementary pattern 3′-miRNA sequence-5′**	**InterPro description of targets**
		**↓**1767(9/10)	B3 DNA binding domain Aux/IAA-ARF-dimerisation DNA-binding pseudobarrel domain AUX/IAA protein
Si005991m	1756-1776	5*'*-AGGCAUACAGGGAGCCAGGCA-3*'*	
sit-miR160		3*'*-**A**CCGUAUGUCCCUCGGUCCGU-5*'*	
		**↓**1364(9/10)	AUX/IAA protein B3 DNA binding domain Auxin response factor Aux/IAA-ARF-dimerisation DNA-binding pseudobarrel domain
Si016509m	1353-1373	5*'*-AGCCAUACAGGGAGCCAGGCA-3*'*	
sit-miR160		3*'*-**A**C*C*GUAUGUCCCUCGGUCCGU-5*'*	
		**↓**1328(6/10)	AUX/IAA protein B3 DNA binding domain Auxin response factor Aux/IAA-ARF-dimerisation DNA-binding pseudobarrel domain
Si034525m	1317-1337	5*'*-AGGCAUACAGGGAGCCAGGCA-3*'*	
sit-miR160		3*'*-**A**CCGUAUGUCCCUCGGUCCGU-5*'*	
		**↓**1085(7/10)	Transcription factor GRAS
Si016508m	1074-1094	5*'*-GAUAUUGGCGCGGCUCAAUCA-3*'*	
sit-miR171a		3*'*-CUAUAACCGCGCCGAGUUAGU-5*'*	
sit-miR171b		3*'*-CUAUAACCG**U**GCCGAGUUAGU-5*'*	
		**↓**1082(2/10)	Transcription factor GRAS
Si016508m	1071-1091	5*'*-AGAGAUAUUGGCGCGGCUCAA-3*'*	
nov-sit-miR15		3*'*-UCUCUAUAACCGCGCCGAGUU-5*'*	
nov-sit-miR14		3*'*-**C**CUCUAUAAC**U**GCGCCGAGUU-5*'*	
nov-sit-miR49		3*'*-UC**A**CUAUAACCG**A**GCCGAGU-5*'*	

Mapping of mRNA cleavage sites by RNA Ligase-Mediated 5′ RACE. The complementary pattern of miRNA sequences and partial sequences of the target mRNAs are shown in the table. All predicted mismatch base parings are shown in bold letters. The positions inferred as 5′ ends of miRNA-guided cleavage products, as revealed by 5′ RACE, and the numbers of sequenced 5′ RACE clones corresponding to each site are indicated by vertical arrowheads.

About 79% (351) of the predicted target genes had functional annotations in InterPro, the integrated resource for protein families, domains, and functional sites (Additional file 6). A large proportion of the predicted targets were annotated as transcription factors; for example, the AP2/ERF domain, homeodomain, and CCAAT-binding transcription factors. In addition, some of the predicted targets were annotated as being involved in various classes of molecular functions, including binding proteins (DNA-binding, RNA-binding, ATP-binding and protein-binding), calcium ion transport proteins, and enzymes (protein kinase, synthetase/ligase, acetate-CoA ligase, glycoside hydrolase and oxidoreductase). The functions of predicted miRNA targets indicated that the miRNAs may play multiple roles in gene regulation networks.

**Figure 5:**
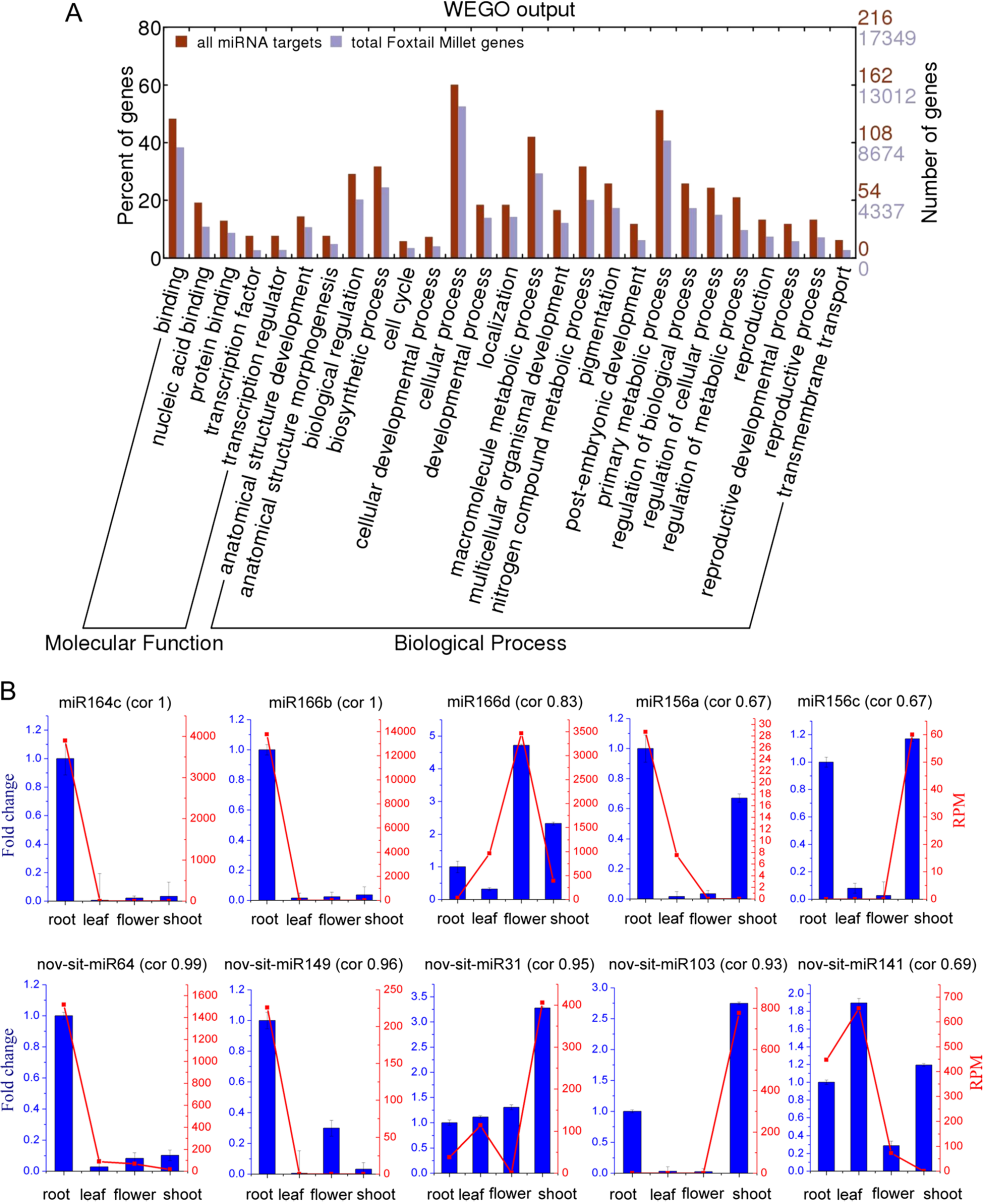
**GO functional enrichment analysis for the predicted target genes of the foxtail millet miRNAs and validations of the smRNA-seq results. (A)** GO functional enrichment analysis for the target genes was compared with the classification for all the foxtail millet genes retrieved from the Phytozome database. **(B)** Stem-loop RT-PCR validation of the smRNA-seq results from foxtail millet (*Setaria italica*). The RT-qPCR expression profiles (blue bars) match the smRNA-seq data (red lines) closely for ten miRNAs we tested. The correlation value (cor) was calculated using Pearson’s product–moment correlation.

To better understand the functional roles of the predicted miRNA target genes in foxtail millet, we analyzed the functional enrichment for all the miRNA targets by gene ontology (GO) [46,47]. The miRNA predicted targets showed enrichment in GO terms from the molecular function and biological process categories, while no enrichment in GO terms was observed in the cellular component category. We found 22 GO terms in the biological process category that showed strong enrichment in biosynthetic process, cellular process, macromolecule metabolic process, regulation of metabolic process and regulation of cellular process. In the molecular function category, the enriched GO terms included binding, nucleic acid binding, and protein-binding. The GO enrichment analysis showed that the predicted targets of the miRNAs were involved in a wide range of regulatory functions as well as some specific biological processes like metabolism, biosynthesis, and gene expression/transcription (Figure 5A).

To analyze whether or not miRNAs in different tissues have different functions, GO enrichment analyses were conducted for the predicted miRNA targets in each of the four tissues. We found that the functions of the targets were similar in all four tissues; the targets were all enriched in biological regulation, biosynthetic process, macromolecule metabolic process and cellular process. In addition, some of the predicted targets had annotations in the cellular component category, including cell, and cell part in leaf and flower. In the root dataset, the target genes were annotated with significantly fewer GO terms than the target genes in the other three tissues. Details of the GO annotations for the target genes in the four tissues are available in Additional file 8.

To confirm the accuracy and reliability of the smRNA-seq results, we randomly chose ten of the miRNAs (five known miRNAs and five novel miRNAs) for stem-loop RT-PCR validation (Figure 5B). The results showed that the stem-loop RT-PCR expression profiles of most miRNAs match the smRNA-seq data closely. While there were three miRNAs expression profiles (miR156c, miR156a and nov-sit-miR141) that were not very consistent.

### Conservation and synteny with sorghum

Previous reports in *Arabidopsis*[48] and in rice [49] have shown that miRNA gene families evolved from segmental, tandem repeat and whole-genome duplication events. A detailed description of the fate of miRNA genes after whole-genome duplication in maize and sorghum has been published [46].

To date, miRNAs expression and function has been well studied in the major food and feed crops, such as sorghum, maize and rice [46,50-53]. Foxtail millet shares a common ancestor with sorghum and maize (~26 Myr ago). This ancestry is more recent than with rice (~34 Myr ago) [54]. Sorghum has experienced one whole genome duplication and maize was experienced two whole genome duplications since they diverged (~13 Myr ago) [55]. To investigate the evolution of foxtail millet miRNAs, we used sorghum as the reference comparison strain. The syntenic analysis was performed between the foxtail millet and sorghum genes.

**Figure 6:**
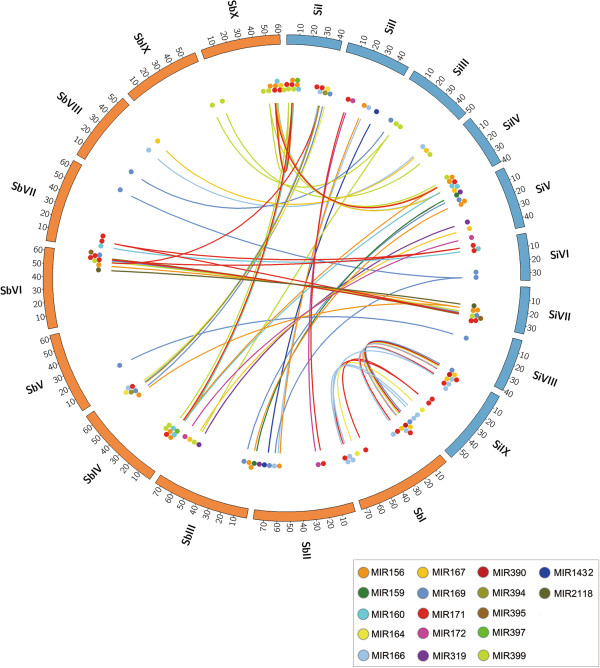
**Comparative map between the foxtail millet and sorghum genomes showing links between syntenic pre-miRNA sequences.** “Sb” represents *Sorghum bicolor* and “Si” represents foxtail millet (*Setaria italica*). Positions of annotated MIR genes are shown using circles that are color-coded according to the family in *Sorghum bicolor*. Links show synteny between MIR genes.

Based on the total number of genes in foxtail millet and in sorghum, we found 919 syntenic blocks containing 28,510 pairs of syntenic genes between the two species. In total, 20,090 foxtail millet and 19,877 sorghum genes, and 65 foxtail millet and 57 sorghum pre-miRNAs, were found within the syntenic regions. Nearly all the pre-miRNAs were located in only 47 (5%) of the 919 syntenic blocks, but within these 47 blocks there were 12,757 syntenic gene pairs corresponding to 45% of the total number of syntenic genes in the two species. We identified 69 pairs of syntenic pre-miRNAs, and all the foxtail millet and sorghum chromosomes had blocks that contained at least one of the conserved syntenic pre-miRNAs (Figure 6 and Additional file 9). We noted that 18 of the novel miRNAs that we identified in foxtail millet had syntenic pre-miRNA sequences and conserved mature miRNA sequences (Table 2). Therefore, in some respects, the 18 foxtail millet miRNAs are new members of already known miRNA families. For example, nov-sit-miR58 and nov-sit-miR60 could be considered to be new members of the miR166 family; similarly, nov-sit-miR14, nov-sit-miR49 and nov-sit-miR50 are new members of the miR171 family.

**Table 2:** Mature sequence and precursor sequence conservation of novel miRNA

**miRNA precursor**	**miRNA sequence**	**Conserved precursor**	**Conserved miRNA family**
nov-sit-MIR105	GCTCACTCCTCTTTCTGTCAGC	sbi-MIR156a/e	miR156
nov-sit-MIR103	GCTCACTTCTCTGTCTGTCAGC	sbi-MIR156f	miR156
nov-sit-MIR16	TTGACAGAAGAGAGCGAGCAC	sbi-MIR156a	miR156
nov-sit-MIR117	CGTGCTCACTACTCTTTCTGTC	sbi-MIR156g	miR156
nov-sit-MIR60	TCGGACCAGGCTTCATTCCCCA	sbi-MIR166b	miR166
nov-sit-MIR05	TTTCGGACCAGGCTTCATTCC	sbi-MIR166c	miR166
nov-sit-MIR58-2	TCGGACCAGGCTTCATTCCCCT	sbi-MIR166a/j	miR166
nov-sit-MIR58-3	TCGGACCAGGCTTCATTCCCCT	sbi-MIR166j	miR166
nov-sit-MIR77-2	TAGCCAAGAATGACTTGCCT	sbi-MIR169i	miR169
nov-sit-MIR32	TTAGCCAAGAATGGCTTGCCTA	sbi-MIR169q	miR169
nov-sit-MIR14	TTGAGCCGCGTCAATATCTCC	sbi-MIR171h	miR171
nov-sit-MIR49	TGAGCCGAGCCAATATCACT	sbi-MIR171c	miR171
nov-sit-MIR50	TGAGCCGAACCAATATCACTC	sbi-MIR171e/f	miR171
nov-sit-MIR148	AGTGGATGGCGCGGGAGCTAA	sbi-MIR319b	miR319
nov-sit-MIR114	CTGAAGTGTTTGGGGAACTC	sbi-MIR395a	miR395
nov-sit-MIR123	CGCCAAAGGAGAATTGCCCTG	sbi-MIR399b/h	miR399
nov-sit-MIR101	GGCAGCTCTCCTCTGGCAGG	sbi-MIR399d	miR399
nov-sit-MIR89	GTGCGGTTCTCCTCTGGCATG	sbi-MIR399h	miR399

## Discussion

Some miRNAs are expressed only in a specific tissue or at a specific site, and an understanding of their unique expression pattern may help in discovering the function of a particular miRNA. To assess the tissue specificity of the miRNAs in our datasets, we investigated the expression levels of the miRNA in the four foxtail millet tissues (leaf, flower, root and shoot). We found that the expression of the sit-miR172 family was higher in the adult leaf and flower datasets and lower in the young shoot dataset. In maize, the miR172 that is responsible for the transition from juvenile to adult was reported to have the same expression pattern [46]. In addition, compared with the other sit-miRNA families, the sit-miR166 family was highly expressed, especially in leaf (over 20, 285 RPM). In maize, the target gene of miR166 is *rolled leaf1*, which can regulate leaf morphogenesis [17,56]. Similarly, in foxtail millet, the target gene of sit-miR166 was predicted as Si034251m, a homolog of *rolled leaf1*. This finding suggests that there may be a similar mechanism of leaf morphogenesis in foxtail millet as the one reported in maize.

We found that, except for a few of the sit-miRNAs that had relatively high expression levels, most of the sit-miRNAs, especially the nov-sit-miRNAs, had low expression levels in the four studied tissues. It has been suggested that the normal physical functions of an organism require various miRNAs working together for precise regulation and control rather than individual miRNAs working separately [57]. If this suggestion is correct, then the miRNAs with low expression might have an effect on the growth of foxtail millet as great as the miRNAs with high expression. A previous report indicated that most of the miRNA targets in maize are conserved across several plant species [46] and we found that this was also true in foxtail millet. For example, miR156 and miR529 were predicted to target genes that encode SBP-box transcription factors [58-60], and miR164, miR169, miR171, miR172 and miR319 were reported to target No Apical Meristem (NAM) [61,62], CCAAT-binding factor (CBF) [63,64], GRAS transcription factor [65], APETALA2 Ethylene-Responsive Element Binding Proteins (AP2-EREBP) [66,67] and Teosinite branched, Cycloidea, and PCF (TCP) [68,69], respectively. In maize and *Arabidopsis*, miR160 and miR167 were found to target auxin response factor [46,70,71]. In foxtail millet, sit-miR827 had 10 predicted target genes, six were SYG1/Pho81/XPR1 (SPX) proteins, and four were NAD (P)-binding proteins, in agreement with a report that, in maize, miR827 could target NAD (P)-binding and SPX proteins [46]. Most of the predicted target genes of the sit-miRNAs are transcription factors that are known to play important roles in plant growth processes and in the regulation of plant development. This result is also consistent with previous reports in other species [21,72,73].

In *A. thaliana*, the GRF gene family (AtGRF) of proteins that are involved in cell expansion in leaf and cotyledon tissues [74] are regulated by miR396 [75]. We found that nov-sit-miR64 was conserved to miR396 of *Arabidopsis* and that its predicted target gene contained the same characteristic regions (QLQ (Gln, Leu, Gln) and WRC (Trp, Arg, Cys) domains) as the AtGRF proteins. Compared with other novel miRNAs, the nov-sit-miR64 had a relatively high expression in leaf (88 RPM), suggesting that nov-sit-miR64 may play an important role in leaf; however, nov-sit-miR64 expression was highest in root (1518 RPM) compared with in the other three tissues (less than 90 RPM). These findings suggest that nov-sit-miR64 (or miR396) not only plays an important role in leaf growth, but may also be involved in the development of root. The expression pattern of nov-sit-miR64 was validated by experiment (Figure 5B).

Foxtail millet is considered to be a diploid, tractable model for polyploid biofuel crops like switchgrass (*Panicum virgatum*) and Napier grass (*Pennisetum purpureum*) [26]. The synthesis of cellulose is a complicated process that depends on carbon fixation, sugar metabolism and transit, and fat metabolism [76-78]. In this study, we also find eight potential miRNA targets that may be involved in the biological synthesis of cellulose and seven miRNAs were predicted to have an effect on the regulation of cellulose biosynthesis (Table 3). For example, sit-miR319 potentially targets four different genes involved in carbohydrate metabolic process, suggesting that sit-miR319 might play a central role in sucrose metabolism and carbon fixation. Cellulose synthase mRNA was predicted to be a target of miR-397 in maize [79], and laccase, which is involved in lignin catabolic process in foxtail millet, was one of the predicted targets of sit-miR397.

**Table 3:** MiRNAs potentially targeting biofuel-related biological processes

**miRNA ID**	**Target ID**	**InterPro ID**	**InterPro description**	**GO annotation**
sit-miR169c/e	Si025232m	IPR001220	Legume lectin domain	Carbohydrate binding
sit-miR319	Si022272m Si022273m Si022276m Si022277m	IPR000490	Glycoside hydrolase, family 17	Hydrolase activity, hydrolyzing O-glycosyl compounds , carbohydrate metabolic process
		IPR013781	Glycoside hydrolase, catalytic domain	Catalytic activity, carbohydrate metabolic process, cation binding
sit-miR397	Si001625m	IPR017761	Laccase	Lignin catabolic process, apoplast, hydroquinone: oxygen oxidoreductase activity, oxidation reduction process
nov-sit-miR32	Si025232m	IPR001220	Legume lectin domain	Carbohydrate binding
nov-sit-miR41	Si035473m	IPR001220	Legume lectin domain	Carbohydrate binding
nov-sit-miR77	Si025232m	IPR001220	Legume lectin domain	Carbohydrate binding
nov-sit-miR155	Si020000m	IPR008089	Nucleotide sugar epimerase	Carbohydrate metabolic process, racemase and epimerase activity, acting on carbohydrates and derivatives

The expression pattern of some sit-miRNA families also showed similar trends as the corresponding conserved miRNAs in different plants. For example, the expression levels of sit-miR156, sit-miR164, sit-miR166 and sit-miR167 were comparatively very high, and sit-miR160, sit-miR319, sit-miR390 and sit-miR394 were comparatively very low, in agreement with similar findings in other plants [21,22,80]. Because two of the characteristics (similar expression patterns and conserved targets) of these miRNAs were similar in other plants, we inferred that the regulatory mechanisms and main functions of these miRNA target genes would also be similar, perhaps explaining the similar expression patterns of the conserved miRNAs in different species.

In this work, we examined the expression profiles of ten miRNAs in four tissues, three of which appeared to not be very consistent with the smRNA-seq data, suggesting that preprocessing and normalizing the data to help in identifying differentially expressed genes is a process that continues to be developed.

## Conclusion

By smRNA sequencing, we identified 43 known miRNAs and 172 novel miRNAs in foxtail millet and studied their expression profiles in four different tissues. Potential targets were predicted with strict criteria as described, and four targets were validated by 5′RACE. The functional annotation provided a deeper understanding of the transcription and regulation of the target genes, confirmed many known regulatory mechanisms, and provided a window into many more potentially novel pathways. Furthermore, a comparative genomic analysis with sorghum contributed to understanding the evolutionary dynamics of miRNA family expansions and will serve as the basis for future scomparative functional genomic studies using syntenic analysis. The identification and characterization of miRNAs from foxtail millet will aid in further research on foxtail millet and other species in the Poaceae family of grasses.

## Methods

### Plant material and sequencing

Foxtail millet inbred line Yugu1 [26,27] was used in this study. Two smRNA libraries were constructed. The smRNAs extracted from 50 shoots of 14-day-old seedlings were used for each library. Seeds of Yugu1 were germinated on moist paper and incubated at 28°C for 24 h, then transferred to pots filled with 1:1 mix of nutrient soil:vermiculite and grown for 9 days in an illuminating incubator (28°C day/20°C night, 14-h photoperiod, 70% relative humidity). The roots of the seedlings were gently washed, then transferred to 1/4 Hoagland’s solution and allowed to grow for an additional 5 days. Briefly, total RNA was isolated using TRIzol reagent (Invitrogen, USA). SmRNA library construction was carried out with an Illumina TruSeq Small RNA sample prep kit (Illumina, USA) according to the manufacturer’s instructions. SmRNA was extracted by running total RNA on a 15% PAGE gel (1× TBE, 7 M urea, 15% acrylamide (19:1 acryl:bis-acryl) in 1% TBE at 200 V for 1 hour and excising the bands in the ~18 to 30 nt size range. 5′ and 3′ adaptors were ligated sequentially to the smRNAs and then amplified by RT-PCR. Samples were prepared for sequencing following the manufacturer’s standard protocol (Illumina TruSeq Small RNA sample prep kit) (Illumina, USA) and sequenced on a HighSeq 2000 sequencer (Illumina) to produce 36-bp single reads. The generated raw reads have been deposited in NCBI’s SRA database under accession numbers SRA062827.

### Identification of foxtail millet miRNA and miRNA*

The foxtail millet genome sequences were downloaded from the Phytozome database (Sitalica_164_hardmasked.fa). To find foxtail millet miRNAs, a previously reported workflow was used [21] with a stricter MFEI [MFE/(precursor length) × 100/(G + C)] cutoff value of greater than 0.85 [35] rather than the previous MFEI threshold of 0.15. First, EMBOSS-einverted [81] was used to identify imperfect inverted repeats from the hard masked genome sequences. Then, the inverted repeats were folded by RNAfold [82] and single loop-stem-loop sequences were selected through an in-house developed Perl script. By checking the secondary structure and copies (less than 10) of these single loop-stem-loop sequences, we identified the miRNA precursor candidates.

Then, for each sequenced RNA sample, short reads were first mapped to all the candidate precursors. The location of the reads was checked to identify and eliminate the reads that mapped on the loop region of the corresponding precursor. Finally, the most abundant reads that were 20 ~ 22 nt in length with no less than 5 reads were regarded as potential miRNAs. A split-screen view of read alignments from a shoot (14-day-old) sample displaying regions of ten miRNA precursors is shown in Additional file 10. These results were displayed using Integrative Genomics Viewer (IGV) [83].

To identify known and conserved miRNAs in foxtail millet, BLASTN was used to match the candidate miRNAs to the mature miRNAs from all 14 of the previously investigated plant species [26] in miRBase Release 20 [84]. The miRNAs that had perfect matches with no mismatches and the same length as the mature plant miRNAs in miRBase were defined as known miRNAs in foxtail millet (sit-miRNAs). The miRNAs that did not have complete matches but had less than three mismatches were defined as conserved miRNA (nov-sit-miRNAs).

To identify miRNA*, the first step of our pipeline is using BLASTN to align smRNAs from our sequencing libraries to miRNA sequences. We identified miRNA* candidates by allowing no more than four mismatches between the smRNA sequences and the miRNA sequences. Then, miRNA precursors were folded by RNAfold and the predicted miRNA* sequences that have 2 nt 3′ overhangs to the mature miRNAs were selected by an in-house developed Perl script, which can be obtained by request. Finally, if the sequence of predicted miRNA* can be found among the miRNA* candidates in the BLASTN output, we defined it as miRNA* in our study.

### Identification of mirtrons in foxtail millet

To find mirtrons in the foxtail millet, we obtained all the intron sequences from the annotation (Sitalica_164_gene.gff3 and Sitalica_164_gene_exons.gff3) and assembly (Sitalica_164_hardmasked.fa) files (data downloaded from Phytozome) files in the Phytozome database. Because the lengths of plant pre-miRNA are usually between 60 ~ 500 nt, we selected the introns within this length range for secondary structure prediction using RNAfold [82]. Sequences that formed a single stem-loop with 0 to 3 nt overhangs at both ends and with a MFE of less than -25 [13] were retained for further filtering. All the smRNA reads from four foxtail millet tissues (leaf, flower, root, shoot) were mapped onto these introns and if the reads can perfectly mapped to ends of the introns were considered as one of the mature mirtron candidates [14].

### Target gene prediction and gene ontology annotation

To predict the target genes of foxtail millet miRNAs more precisely, the foxtail millet gene set (release 164) and annotation data were downloaded from Phytozome. There were nearly 392,553 entries in the transcript sequence file. We used a target prediction method with a modified scoring system as described previously [38]. Basically, targets should fulfill the following criteria: no more than three mismatches between miRNA and target, a position independent score of no more than 3 [85], a position-dependent penalty score of no more than 4 [86], and MFE ratios for miRNA:target duplexes and miRNA:target-binding site duplexes greater than 0.75 [32]. The InterPro resource [87] was used to assign functional annotations to the predicted target genes. We subjected the potential miRNA targets to a functional enrichment analysis using BGI WEGO [46,88]. The Blast2GO software (v2.5.1) [89] with the default parameters was used to obtain the GO terms for each foxtail millet gene. The WEGO online tool (http://wego.genomics.org.cn/cgi-bin/wego/index.pl) [90] was used to perform a GO enrichment analysis of the miRNA targets. The Pearson Chi-square test was used for statistical analysis. GO categories that show a significant (α =0.05) enrichment were analyzed and displayed in the output histogram of the WEGO figure.

### MiRNA target validation by 5′RACE

cDNA templates were prepared from the total RNA that was extracted from the 14-day-old shoots using a GeneRacer kit (Invitrogen) according to the manufacturer’s instructions. For each miRNA target gene, two gene-specific primers (GSP1 and NGSP1) were designed using Primer Premier 5.0 software [91]. These two primers were used for two rounds of PCR and the nested PCR products that were obtained were analyzed on a 1% agarose gel. Positive PCR products were cloned into a pMD19-T vector (TaKaRa, Dalian, China) and transformed to *E. coli* DH10B cells. Sequencing was carried out by Majorbio (Shanghai, China). The primers that were used in this study are listed in Additional file 11.

### Validation of novel miRNA by stem-loop RT-PCR

Total RNA from foxtail millet shoot (14-day-old) was extracted as described in miRNA target validation by 5′RACE. PCR was performed as follows: 94°C for 2 min, 40 cycles of 94°C for 15 s and 60°C for 1 min. The PCR products were analyzed by 4% agarose gel and the specificity of the amplification was validated by sequencing the products. The primer design and the stem-loop RT-PCR procedure were performed as described previously [92,93]. All primers used in the study are listed in Additional file 11.

### Quantification of microRNAs by stem-loop RT-PCR

Total RNA was isolated from roots, leaves, flowers and shoots (14-day-old) using TRIzol Reagent (Invitrogen, USA) and digested with RNase-free DNase I (Promega, USA) to remove genomic DNA contamination. About 100 ng of DNA-free total RNA was hybridized with a miRNA-specific stem-loop RT primer. The hybridized miRNA molecules were then reverse transcribed into cDNA as described [92,93]. Real-time PCR was performed using SYBR@ Premix Ex TaqTM (TaKaRa, Japan) on the 7500 Real Time PCR System (Applied Biosystems, USA) (see Additional file 11 for the primer sequences). All reactions were performed in triplicate. The cycling parameters are as follows: 95°C for 30 sec, 40 cycles of 95°C for 3 sec, 60°C for 30 sec. The endogenous reference, U6 [94], was analyzed on the cDNA template converted from total RNA using U6-specific RT primers (Additional file 11). The 2-^ΔΔCT^ method [95] was used to calculate the relative gene expression levels, which were normalized to the expression level of U6. To verify the specificity of the PCR-amplification, some amplicons were cloned and sequenced.

### Synteny analysis of foxtail millet and sorghum

The protein-coding gene sequences of foxtail millet (release 164) and sorghum (release 79) were downloaded from Phytozome. BLASTN (Evalue = 10^-3^) was used to identify homologous protein-coding and pre-miRNA genes between foxtail millet and sorghum. (The sorghum pre-miRNA sequences were downloaded from miRBase release 20) DAGchainer [96] was used to identify collinear chains among the homologs. We used Circos plots [97] to show the collinear relationships between the foxtail millet and sorghum pre-miRNA sequences.

## Abbreviations

AMP1: ALTERED MERISTEM PROGRAM1; AGO: ARGONAUTE; AP2-EREBP: APETALA2 Ethylene-responsive element binding proteins; AtGRF: *A. thaliana,* the GRF gene family; CBF: CCAAT-binding factor; DCL1: DICER-LIKE1; ER: endoplasmic reticulum; GRAS: GAI, RGA, SCR; IGV: Integrative genomics viewer; MFEI: Minimal folding free energies index; miRNAs: microRNAs; mRNA: Messenger RNA; NAM: No apical meristem; nt: nucleotide; pri-miRNAs: primary miRNAs; QLQ: Gln, Leu, Gln; RISC: RNA-induced silencing complex; RNApol II: RNA polymerase II; RNApol III: RNA polymerase III; RPM: reads per million; smRNAs: small RNAs; SPX: SYG1/Pho81/XPR1; TCP: Teosinite branched, Cycloidea and PCF; WRC: Trp, Arg, Cys.

## Competing interests

The authors declare that they have no competing interests.

## Authors’ contributions

JY and SX conceived and designed the research. FY performed the experimental validation and drafted the manuscript. FY, SX and YL analyzed the data. FY and XQ prepared the samples. JY revised and prepared the final manuscript. All authors read and approved the final manuscript.

## Supplementary Material

Additional file 1 **Statistics of reads for small RNAs in foxtail millet. (A)** Total reads of 18 ~ 31nt small RNAs distribution from four different tissues. **(B)** Overlap among four sequenced small RNA libraries. Click here for file

Additional file 2 Detailed information of known miRNAs in foxtail millet. Click here for file

Additional file 3 Detailed information of novel miRNAs. Click here for file

Additional file 4 Secondary structure of novel miRNA precursors. Click here for file

Additional file 5 The detail information about predicted mirtron candidates. Click here for file

Additional file 6 Potential targets of all miRNAs. Annotations were retrieved from the InterPro database. Click here for file

Additional file 7 The miRNAs (whose targets have been validated) validated by stem-loop RT-PCR and sequencing. Click here for file

Additional file 8 GO functional enrichment analysis for predicted target genes of miRNAs in four tissues respectively with comparisons to total foxtail millet genes. Click here for file

Additional file 9 Syntenic sites harboring MIR genes in foxtail millet and sorghum. Click here for file

Additional file 10 Split-screen view of read alignments from shoot (14-day-old) sample displaying regions of ten miRNA precursors. Click here for file

Additional file 11 Primers used in this study. Click here for file
