# Supplementary material for: Genome-wide characterization of microRNA in foxtail millet (Setaria italica)
Source: BMC Plant Biol. 2013 Dec 13;13:212. doi: 10.1186/1471-2229-13-212 (PMC3878754; doi:10.1186/1471-2229-13-212)

Additional file 1: Statistics of reads for small RNAs in foxtail millet

(A) Total reads of 18~31nt small RNAs distribution from four different tissues.

(B) Overlap among four sequenced small RNA libraries.

A

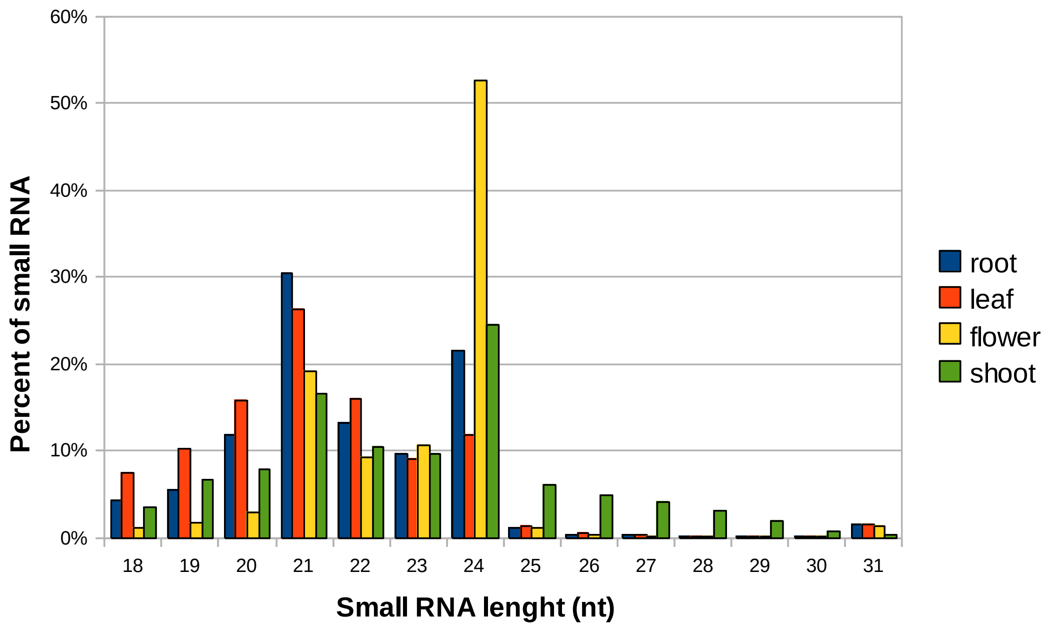

B

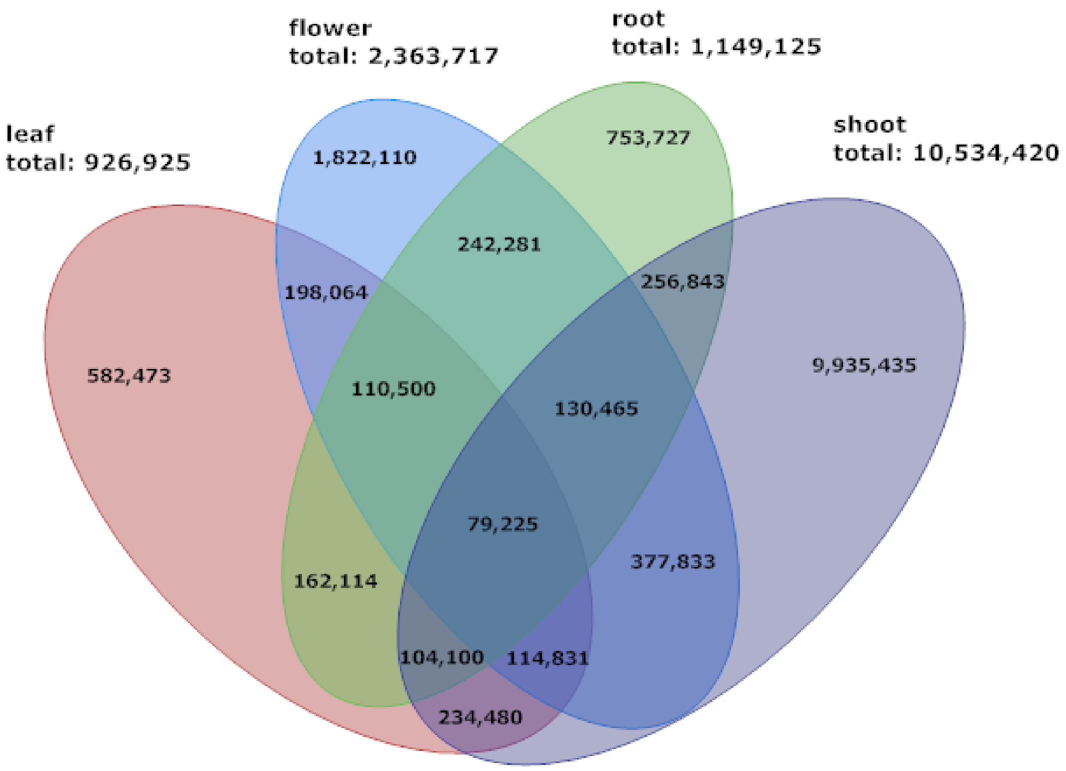

Supplement: Additional file 1 — Statistics of reads for small RNAs in foxtail millet. (A) Total reads of 18 ~ 31nt small RNAs distribution from four different tissues. (B) Overlap among four sequenced small RNA libraries. [file 1471-2229-13-212-S1.pdf]
