# Supplementary material for: Genome-wide characterization of microRNA in foxtail millet (Setaria italica)
Source: BMC Plant Biol. 2013 Dec 13;13:212. doi: 10.1186/1471-2229-13-212 (PMC3878754; doi:10.1186/1471-2229-13-212)

Additional file 4: Secondary structure of novel miRNA precursors

Red colored letter: mature miRNA sequence; blue colored letter: miRNA* sequence, nov-sit-miR01---nov-sit-miR60 which correspond to 75 precursors had been exhibited there.

MIR01

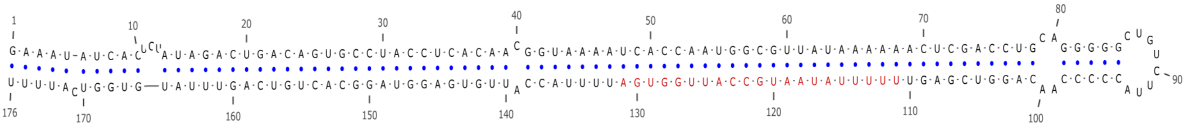

MIR02(*)

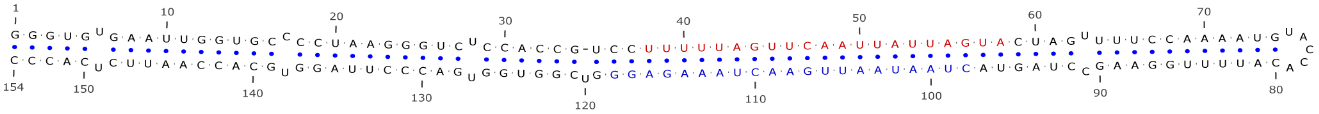

MIR03

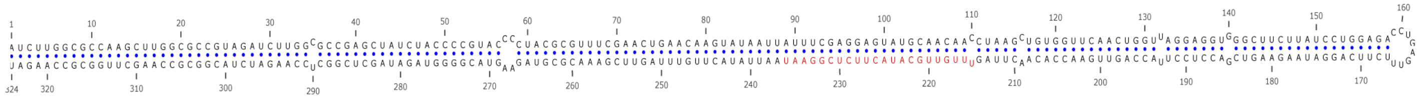

MIR04

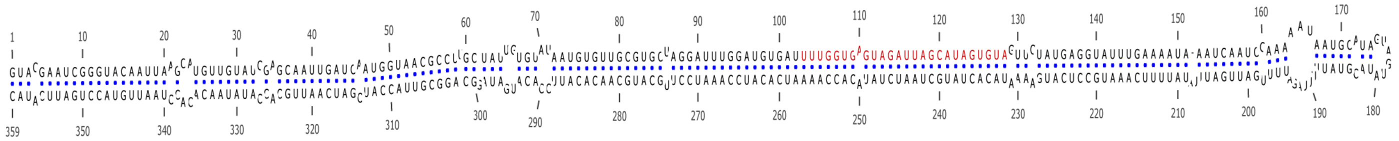

MIR05(*)

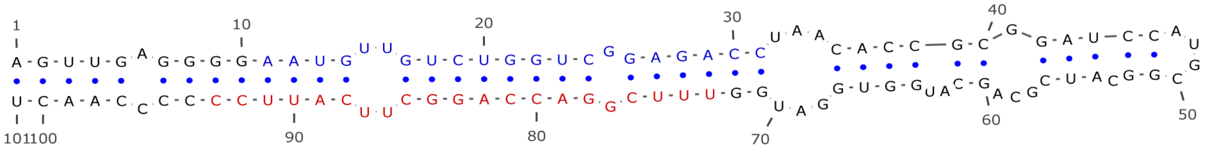

MIR06-1

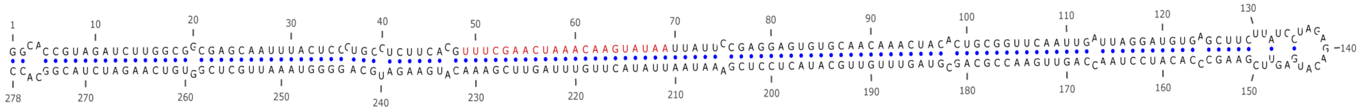

MIR06-2

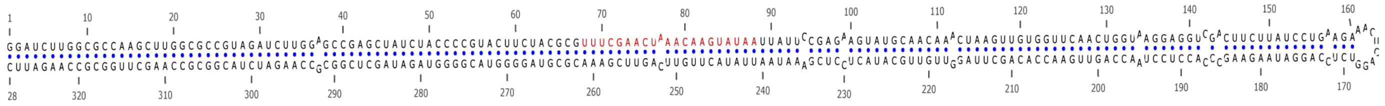

MIR06-3

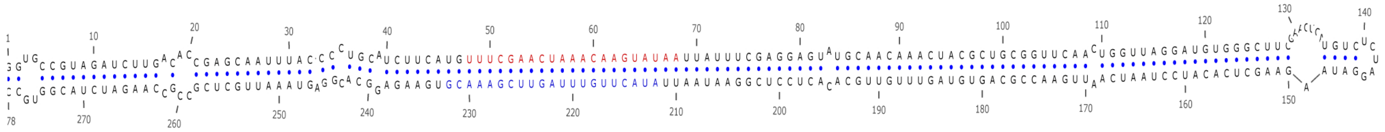

MIR07-1

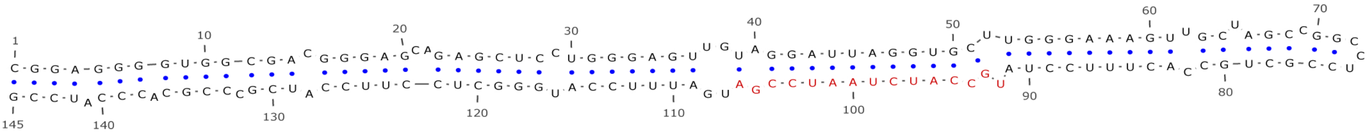

MIR07-2(*)

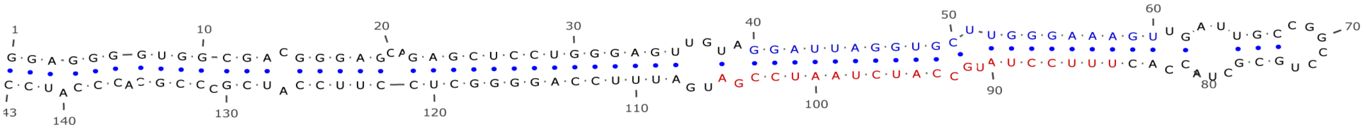

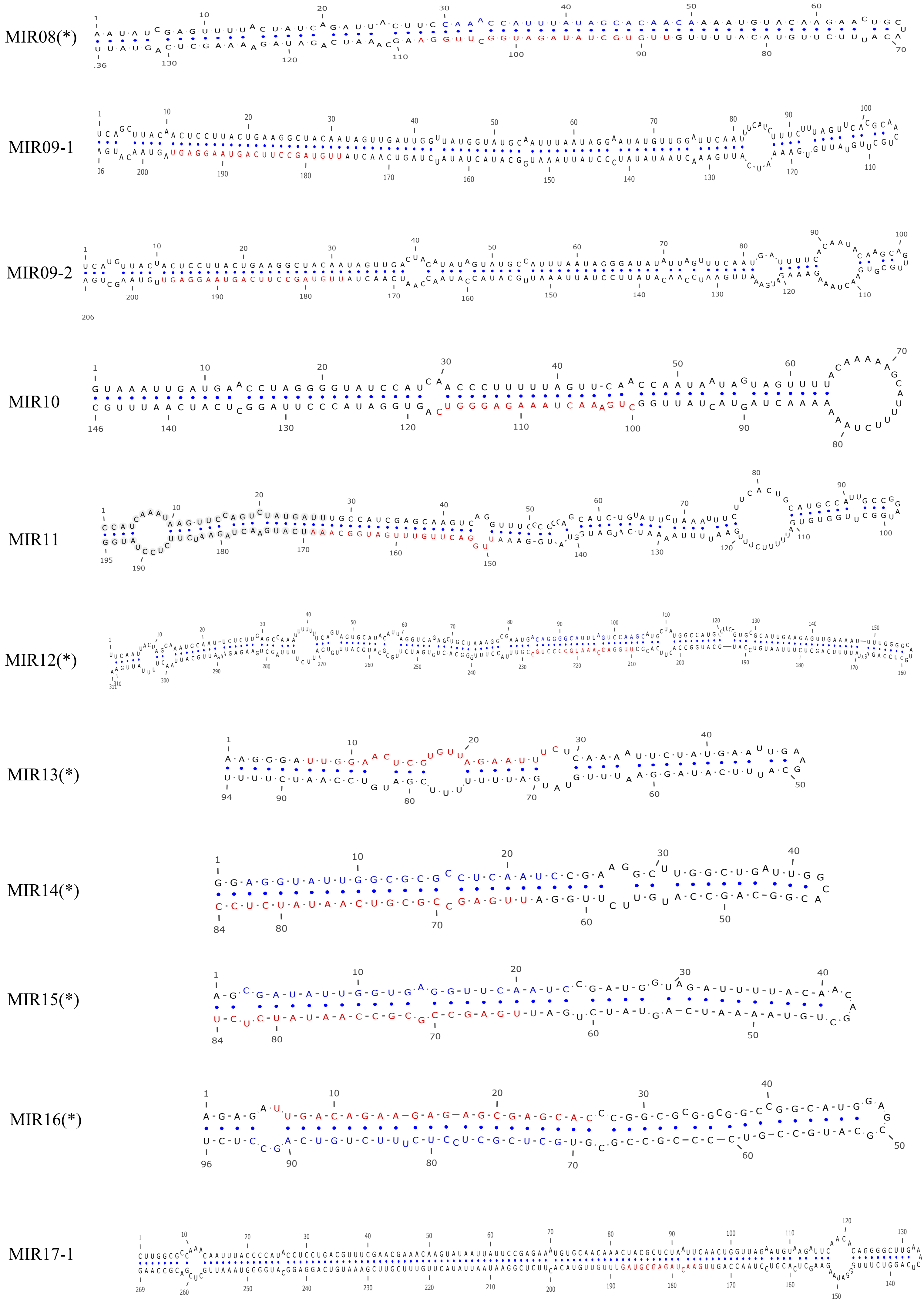
MIR08(*)
MIR09-1
MIR09-2
MIR10
MIR11
MIR12(*)
MIR13(*)
MIR14(*)
MIR15(*)
MIR16(*)
MIR17-1

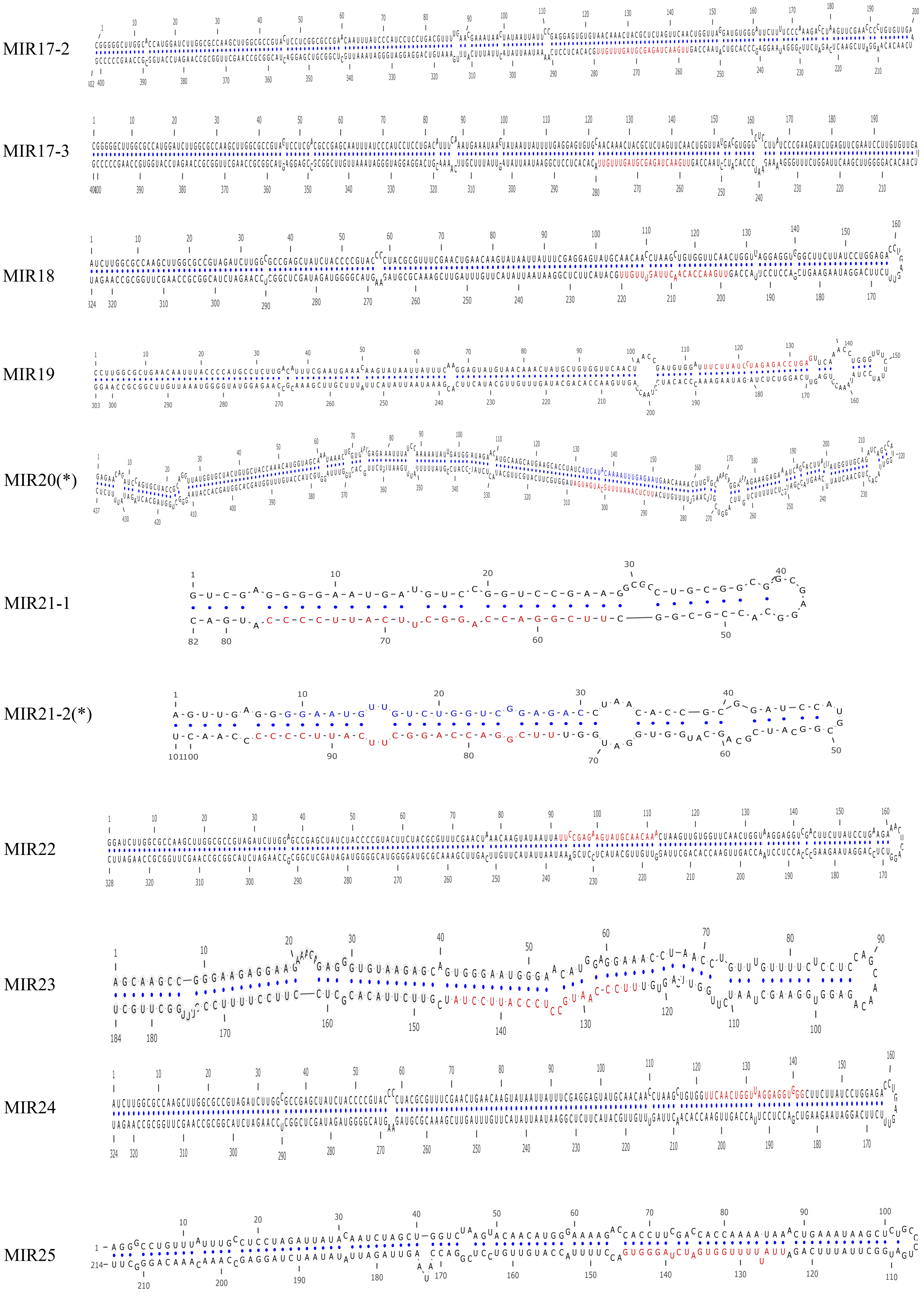
MIR17-2
MIR17-3
MIR18
MIR19
MIR20(*)
MIR21-1
MIR21-2(*)
MIR22
MIR23
MIR24
MIR25

MIR26

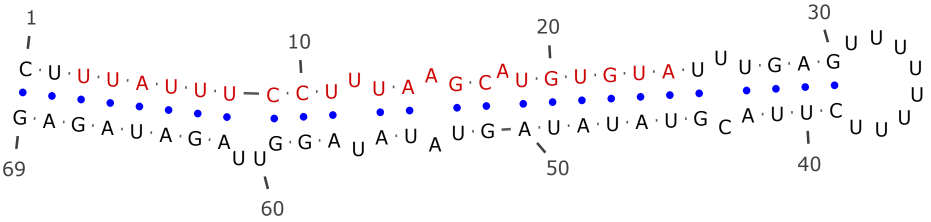

MIR27

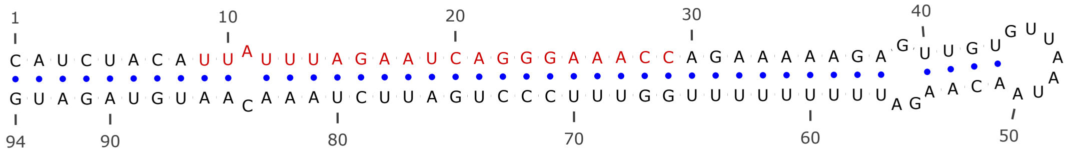

MIR28

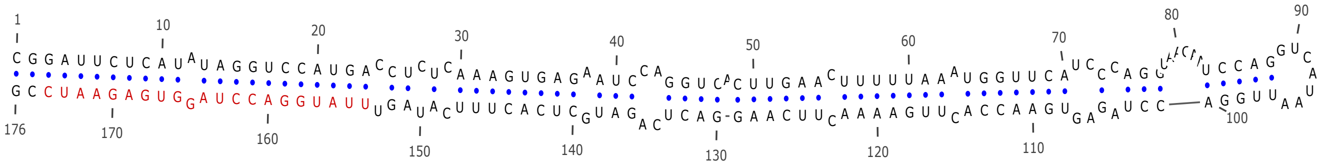

MIR29

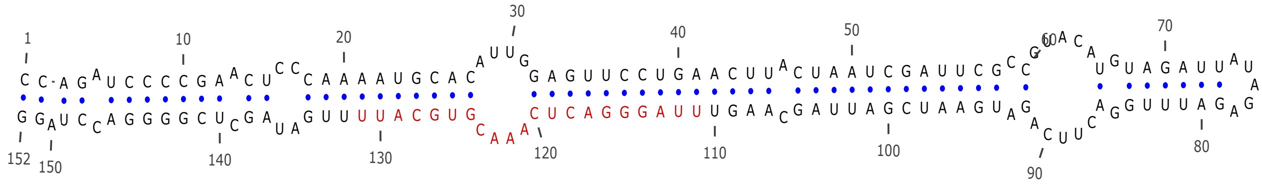

MIR30(*)

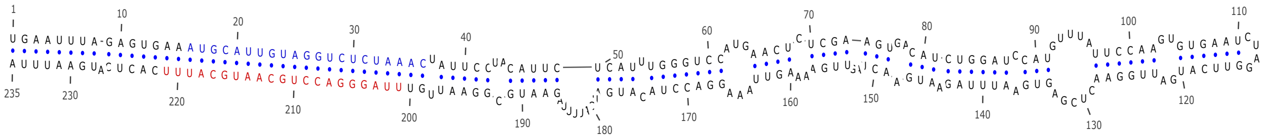

MIR31(*)

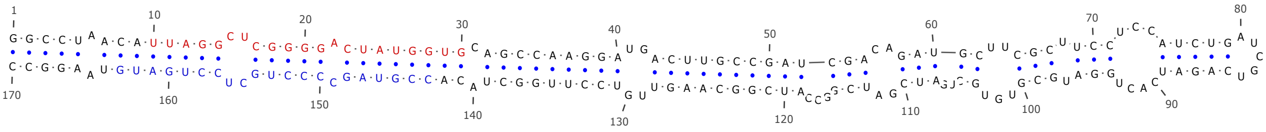

MIR32

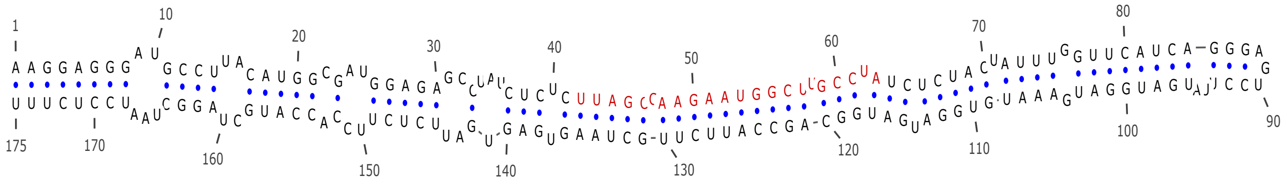

MIR33

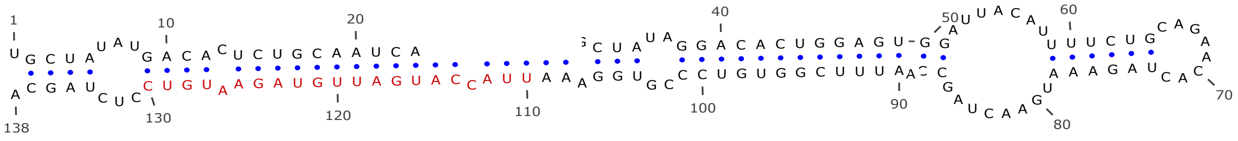

MIR34

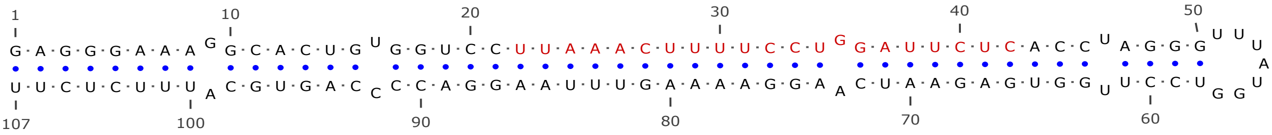

MIR35

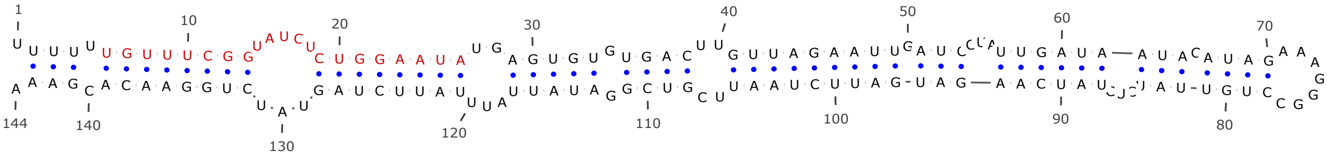

MIR36(*)

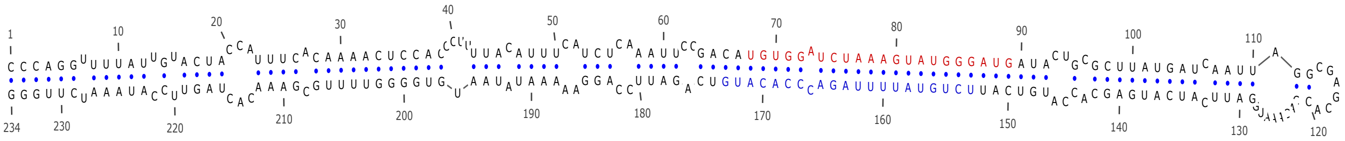

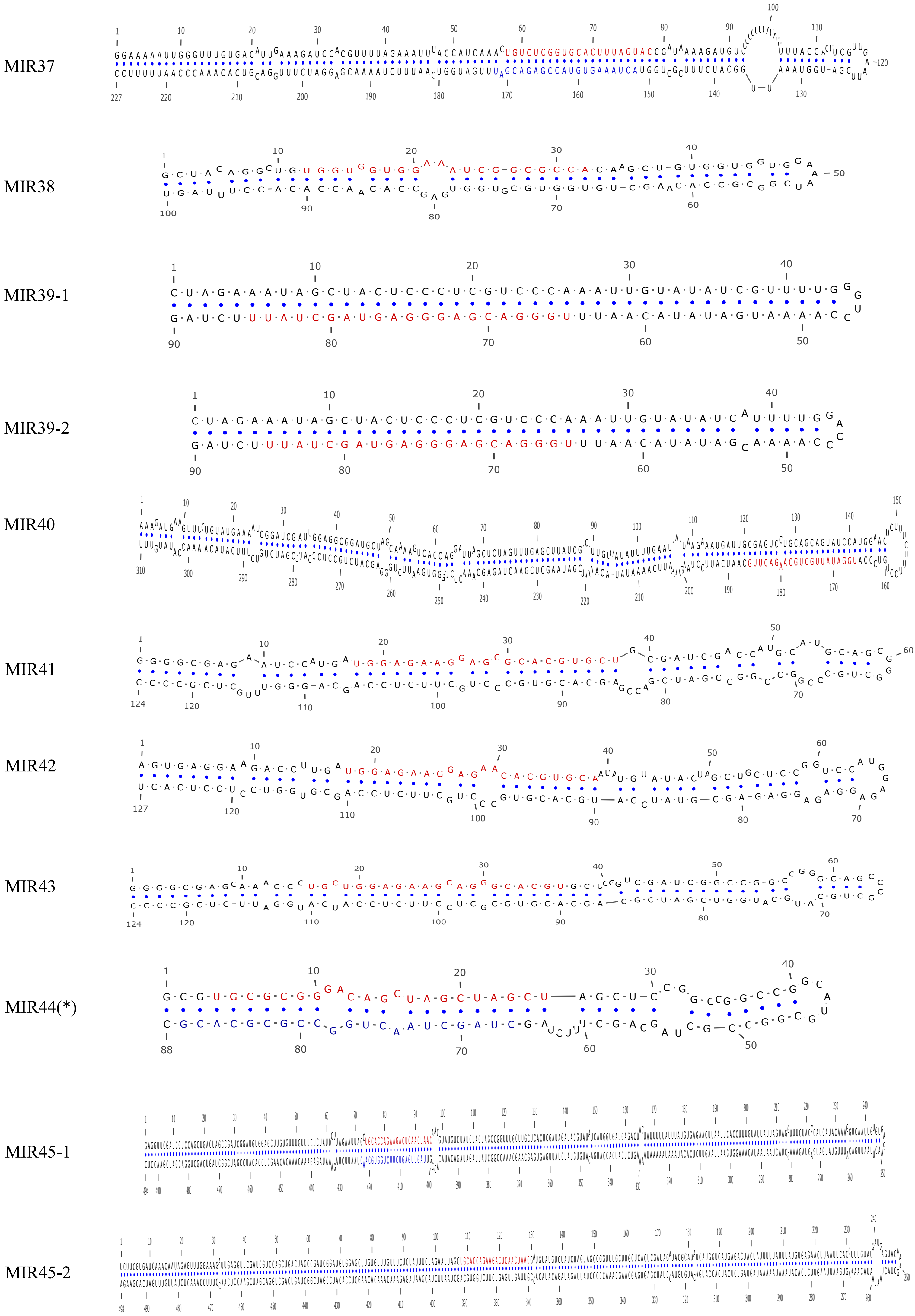
MIR37
MIR38
MIR39-1
MIR39-2
MIR40
MIR41
MIR42
MIR43
MIR44(*)
MIR45-1
MIR45-2

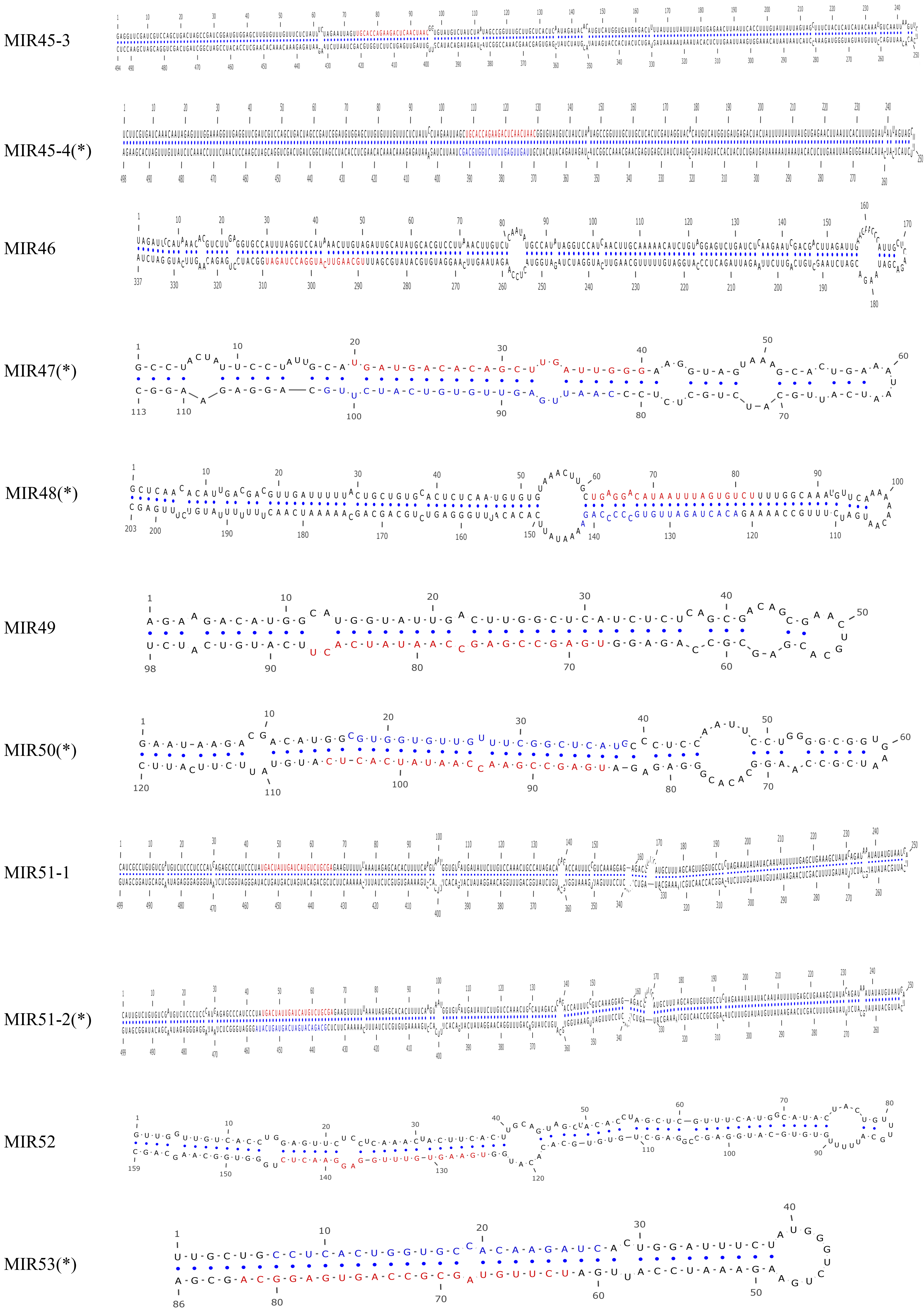
MIR45-3
MIR45-4(*)
MIR46
MIR47(*)
MIR48(*)
MIR49
MIR50(*)
MIR51-1
MIR51-2(*)
MIR52
MIR53(*)

MIR54(*)

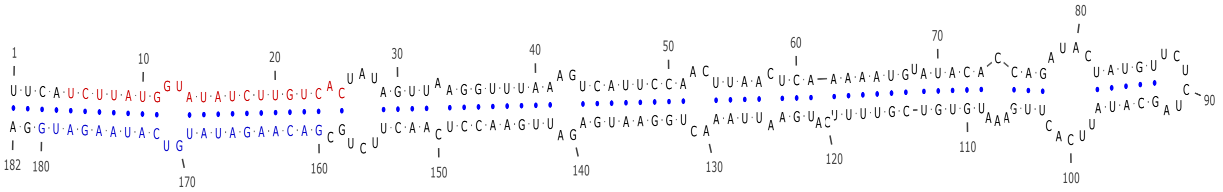

MIR55

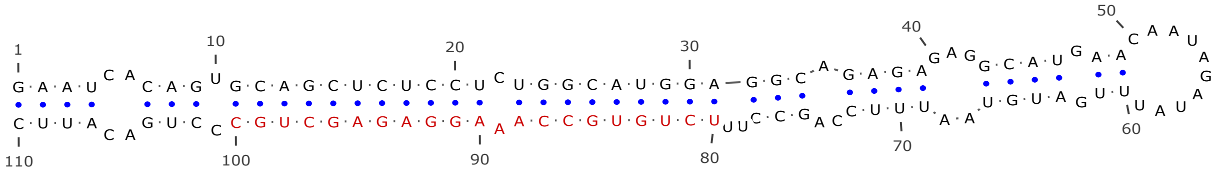

MIR56

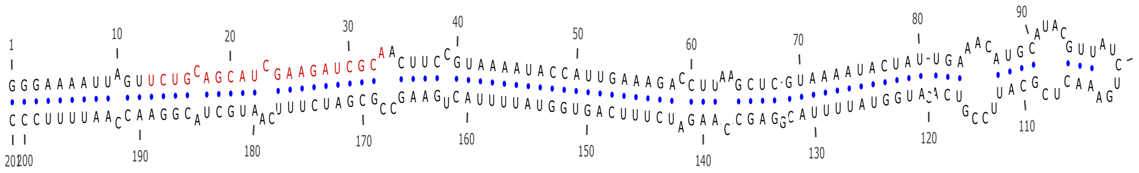

MIR57(*)

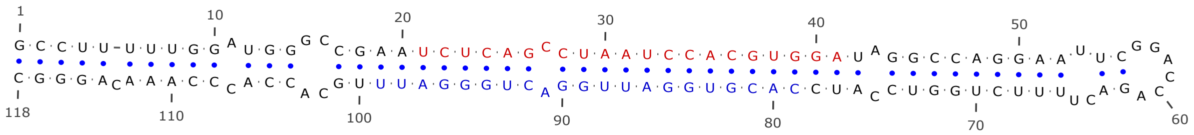

MIR58-1(*)

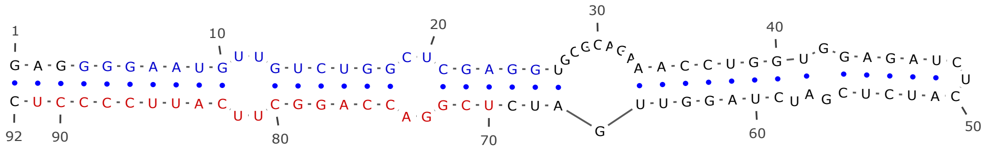

MIR58-2(*)

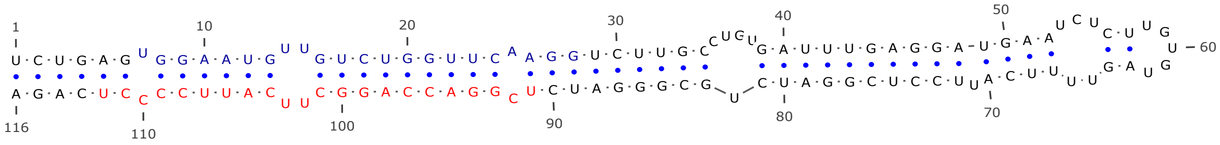

MIR58-3(*)

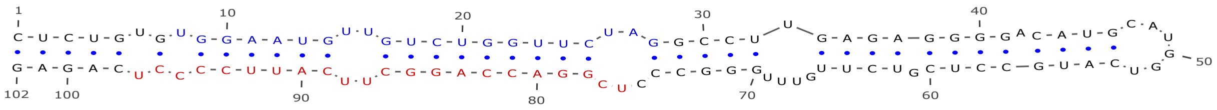

MIR59

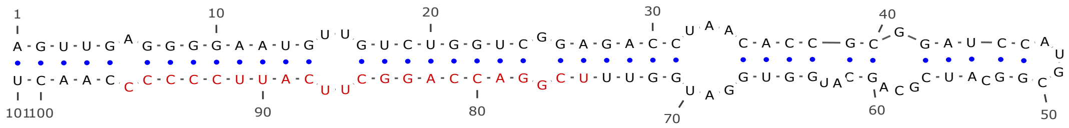

MIR60

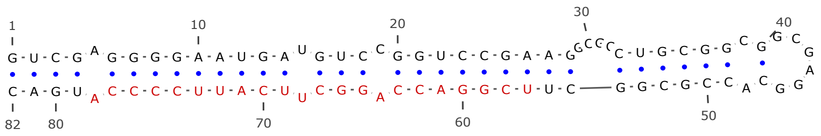

Supplement: Additional file 4 — Secondary structure of novel miRNA precursors. [file 1471-2229-13-212-S4.pdf]
