# Supplementary material for: Genome-wide characterization of microRNA in foxtail millet (Setaria italica)
Source: BMC Plant Biol. 2013 Dec 13;13:212. doi: 10.1186/1471-2229-13-212 (PMC3878754; doi:10.1186/1471-2229-13-212)
Supplement: Additional file 5 — The detail information about predicted mirtron candidates. [file 1471-2229-13-212-S5.pdf]

Additional file5: The detail information about predicted mirtron candidates

The mirtron precursors with aligned small RNAs and their predicted secondary structures were shown. There possible mirtrons were highlighted in red color and the number of reads existed in our small RNA data were shown in the brackets.

1.PAC:19709968

GTGCGTGCCTCACCAATCCAATGCTGGAATCTGGCAATGCCGGAGACCGTACTCTCTAGCCGCGTTCTGAATTTGCTTGGTTTGGCGTCGCAG

GTGCGTGCCTCACCAATCCAATGC(1)

TGAATTTGCTTGGTTTGGCGTCGCAG(1)

TTCTGAATTTGCTTGGTTTGGCGT (1)

TTTGCTTGGTTTGGCGTCGCA(1)

AATTTGCTTGGTTTGGCGTCG(1)

GCGTTCTGAATTTGCTTGGTT(1)

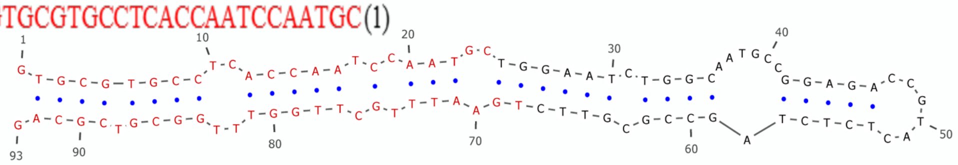

2.PAC:19675165

GTGCGGTCCTCCCTGCCTGCCCTGTTTCCCTCACGAGAAATCCTCCCTTTGGTTGCCCAAATCTGAGATGTTTCCCCTTGAATTGCCGGATTGGTGGTGGGTGGGTGCAG

CCGGATTGGTGGTGGGTGGGTGCAG (7)

CCGGATTGGTGGTGGGTGGGTG(1)

CGGATTGGTGGTGGGTGGGTGCA(1)

CCGGATTGGTGGTGGGTGGGTGC(2)

TTGGTGGTGGGTGGGTGC(1)

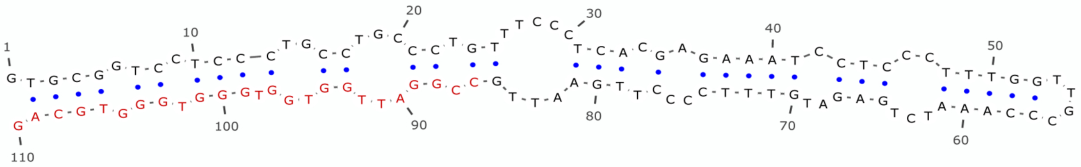

Num	Intron	Chr	Strand	Location	Length
1	PAC:19709968	2	+	8989038-8989130	93
2	PAC:19675165	5	-	33222013-33222122	110
