# Supplementary material for: Genome-wide characterization of microRNA in foxtail millet (Setaria italica)
Source: BMC Plant Biol. 2013 Dec 13;13:212. doi: 10.1186/1471-2229-13-212 (PMC3878754; doi:10.1186/1471-2229-13-212)

Additional file 7: The miRNAs (whose targets have been validated) validated by stem-loop RT-PCR and sequencing

Lanes 1 to 6: miR160, miR171a, miR171b, nov-sit-miR14, nov-sit-miR15 and nov-sit-49, respectively. Lane M, 50 bp DNA ladder. Sequencing results showed we detected miRNA160, miRNA171a, nov-sit-miR14, nov-sit-miR15 and nov-sit-miR49 successfully but failed to detect miRNA171b. RNA used in this experiment is from 14-day-old shoots.

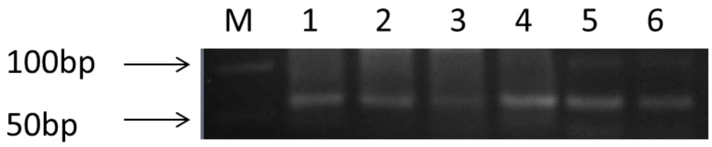

Supplement: Additional file 7 — The miRNAs (whose targets have been validated) validated by stem-loop RT-PCR and sequencing. [file 1471-2229-13-212-S7.pdf]
