# Supplementary material for: Genome-wide characterization of microRNA in foxtail millet (Setaria italica)
Source: BMC Plant Biol. 2013 Dec 13;13:212. doi: 10.1186/1471-2229-13-212 (PMC3878754; doi:10.1186/1471-2229-13-212)
Supplement: Additional file 8 — GO functional enrichment analysis for predicted target genes of miRNAs in four tissues respectively with comparisons to total foxtail millet genes. [file 1471-2229-13-212-S8.pdf]

Additional file 8: GO functional enrichment analysis for predicted target genes of miRNAs in four tissues respectively with comparisons to total foxtail millet genes

(A) GO functional enrichment analysis for predicted target genes of miRNAs in root.

(B) GO functional enrichment analysis for predicted target genes of miRNAs in leaf.

(C) GO functional enrichment analysis for predicted target genes of miRNAs in flower.

(D) GO functional enrichment analysis for predicted target genes of miRNAs in shoot.

A

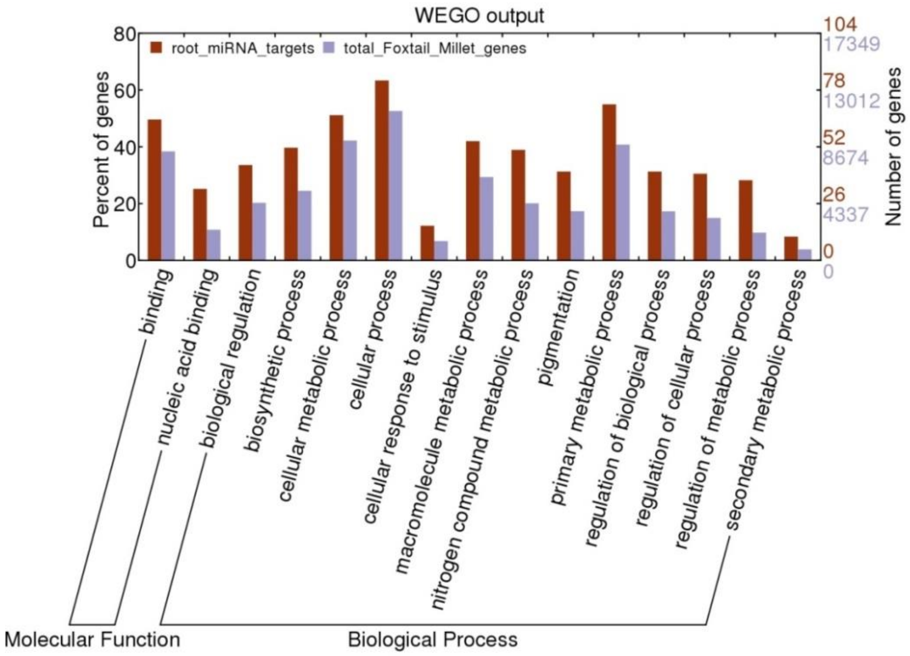

B

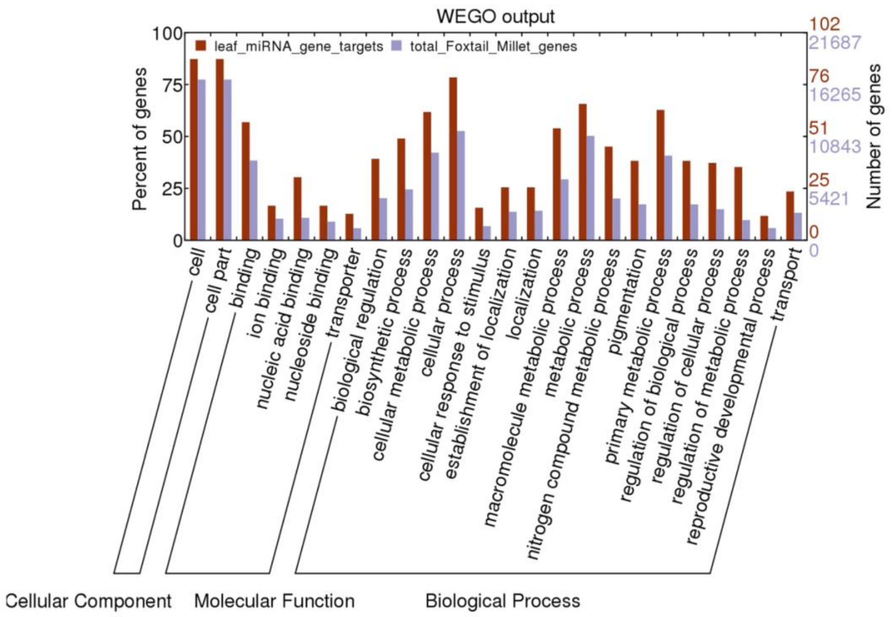

C

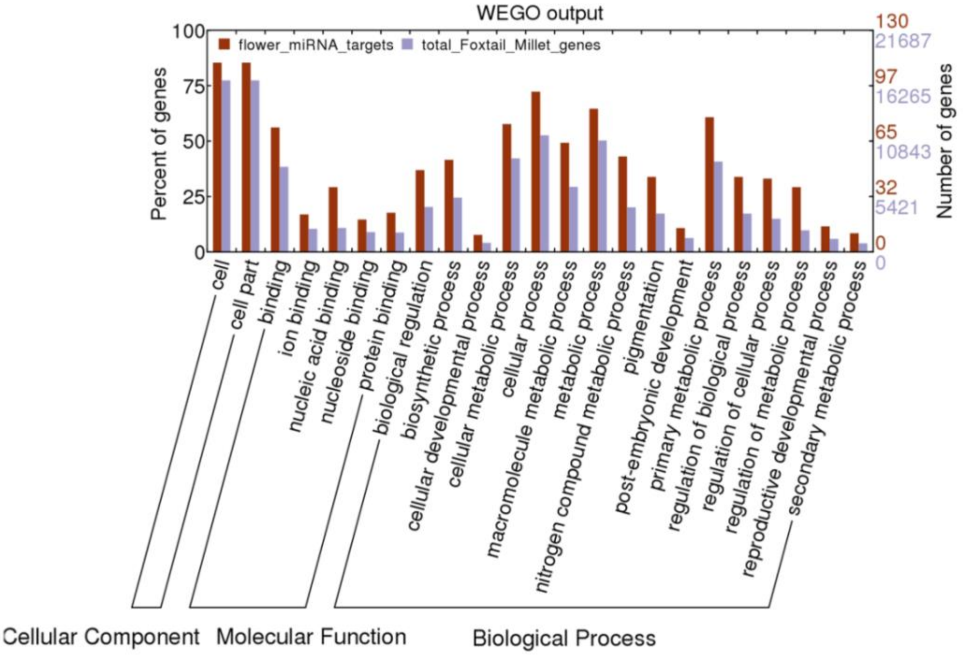

WEGO output
flower_miRNA_targets
total_Foxtail_Millet_genes
Percent of genes
Number of genes
100
75
50
25
0
130
21687
97
16265
65
10843
32
5421
0
0
cell
cell part
binding
ion binding
nucleic acid binding
nucleoside binding
protein binding
biological regulation
biosynthetic process
cellular developmental process
cellular metabolic process
cellular process
macromolecule metabolic process
metabolic process
nitrogen compound metabolic process
pigmentation
post-embryonic development
primary metabolic process
regulation of biological process
regulation of cellular process
regulation of metabolic process
reproductive developmental process
secondary metabolic process
Cellular Component
Molecular Function
Biological Process

D

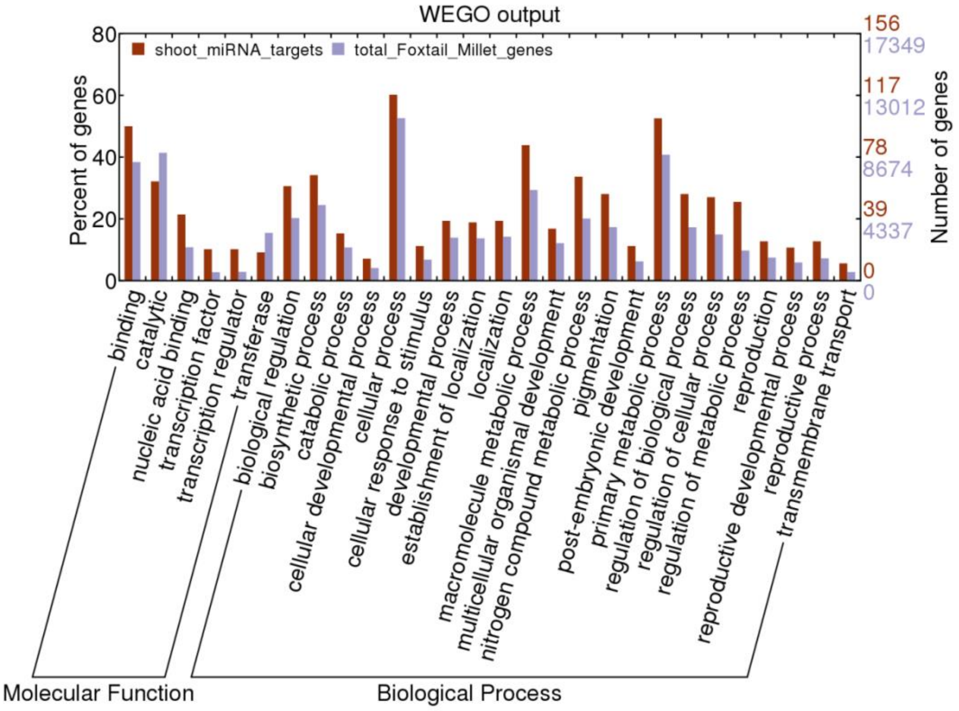

WEGO output
shoot_miRNA_targets
total_Foxtail_Millet_genes
Percent of genes
Number of genes
80
60
40
20
0
156
17349
117
13012
78
8674
39
4337
0
0
binding
catalytic
nucleic acid binding
transcription factor
transcription regulator
transferase
biological regulation
biosynthetic process
catabolic process
cellular developmental process
cellular process
cellular response to stimulus
developmental process
establishment of localization
localization
macromolecule metabolic process
multicellular organismal development
nitrogen compound metabolic process
pigmentation
post-embryonic development
primary metabolic process
regulation of biological process
regulation of cellular process
regulation of metabolic process
reproduction
reproductive developmental process
reproductive process
transmembrane transport
Molecular Function
Biological Process
