# Supplementary material for: Genome-wide characterization of microRNA in foxtail millet (Setaria italica)
Source: BMC Plant Biol. 2013 Dec 13;13:212. doi: 10.1186/1471-2229-13-212 (PMC3878754; doi:10.1186/1471-2229-13-212)
Supplement: Additional file 10 — Split-screen view of read alignments from shoot (14-day-old) sample displaying regions of ten miRNA precursors. [file 1471-2229-13-212-S10.pdf]

Additional file 10: Split-screen view of read alignments from a shoot (14-day-old) sample displaying regions of ten miRNA precursors

In these examples, alignments whose sequence of the reads mapped to the reference genome are color-coded in pink; alignments whose reverse complement sequence of the reads mapped to the reference genome are color-coded in purple.

> nov-sit-miR01

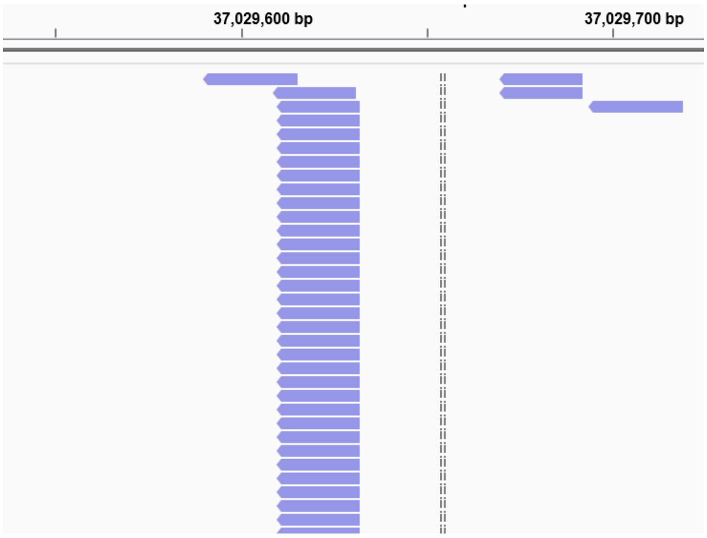

miR01 (93%)

miR01 precursor

scaffold	Position
5	37,029,566-37,029,741(-)

>nov-sit-miR07

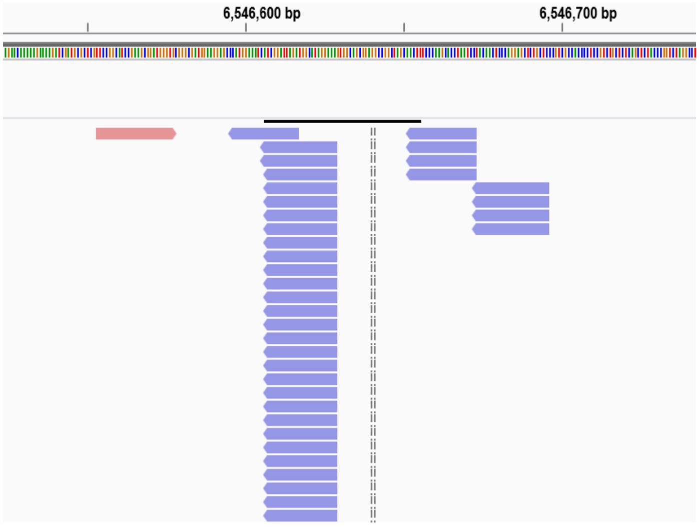

miR07 (99%)

miR07-1 precusor

scaffold	Position
5	6,546,568-6,546,712(-)

>nov-sit-miR08

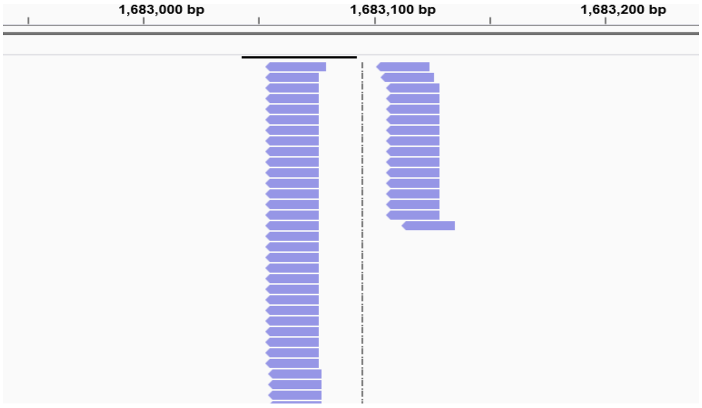

miR08 (87%)

miR08 precusor

scaffold	Position
6	1,683,028-1,683,163(-)

>nov-sit-miR12

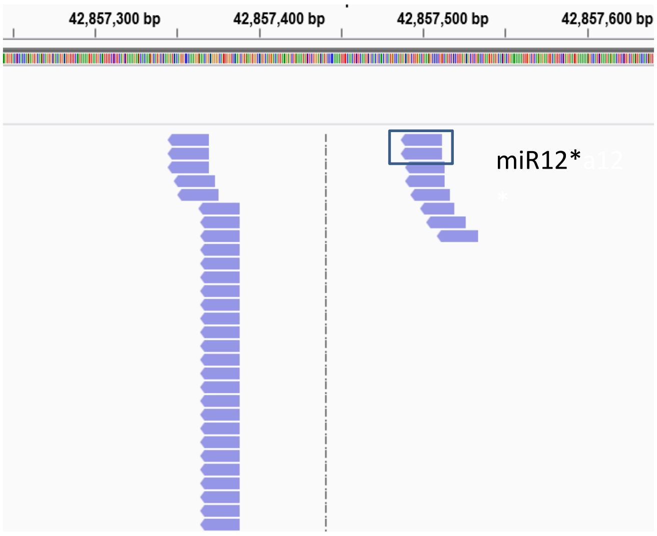

miR12 (78%)

miR12 precursor

scaffold	Position
2	42,857,286-42,857,596(-)

>nov-sit-miR15

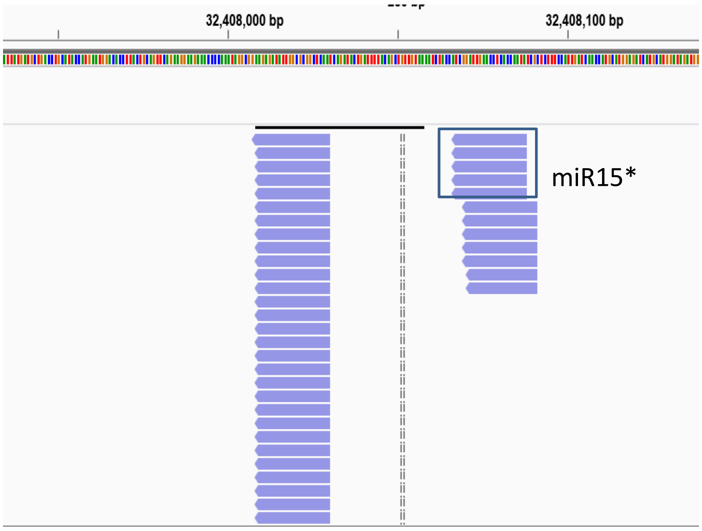

miR15 (96%)

miR15 precursor

scaffold	Position
4	32,408,009-32,408,092(-)

>nov-sit-21

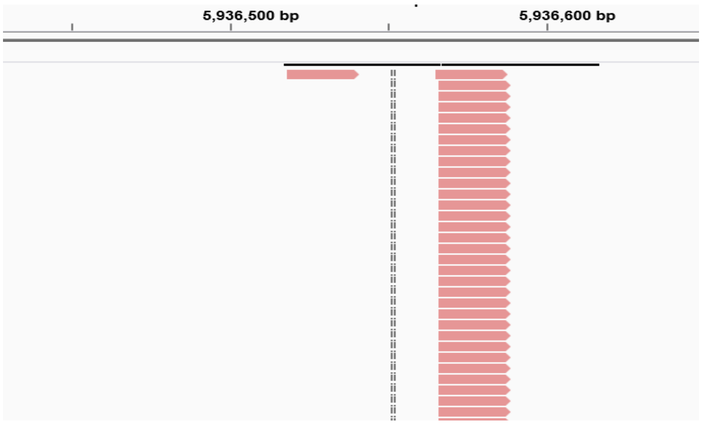

miR21 (99%)

miR21-1 precursor

scaffold	Position
9	5,936,510-5,936,591(+)

>nov-sit-miR25

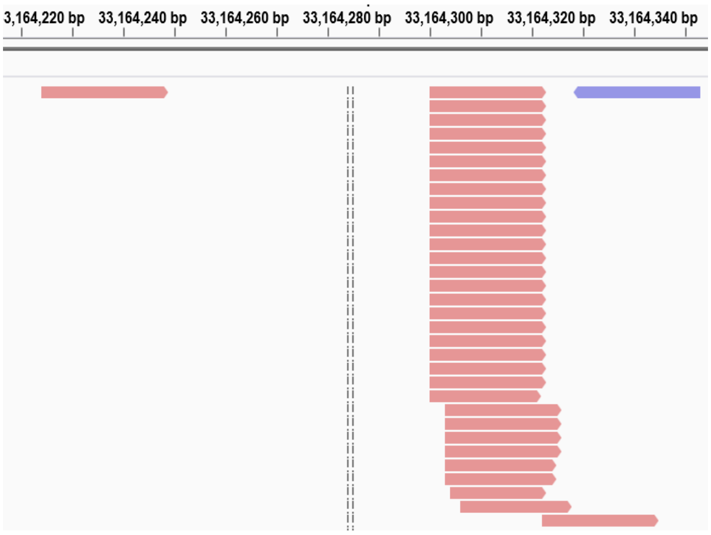

miR25 (91%)

miR25 precursor

scaffold	Position
8	33,164,177-33,164,390(+)

>nov-sit-miR33

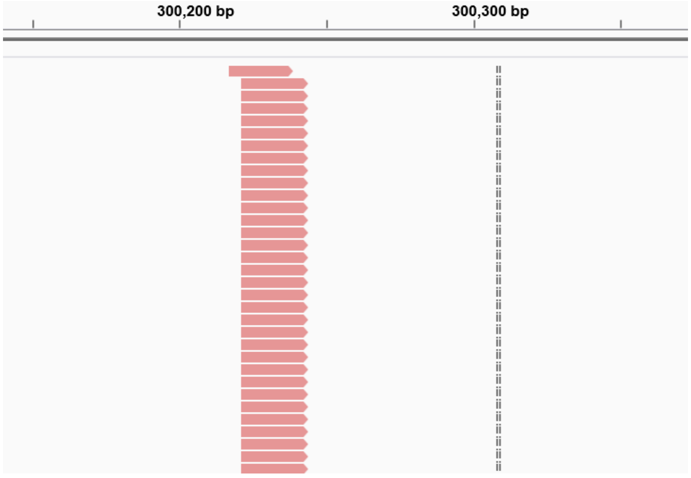

miR33 (100%)

miR33 precursor

scaffold	Position
9	300,112-300,249(+)

> nov-sit-miR36

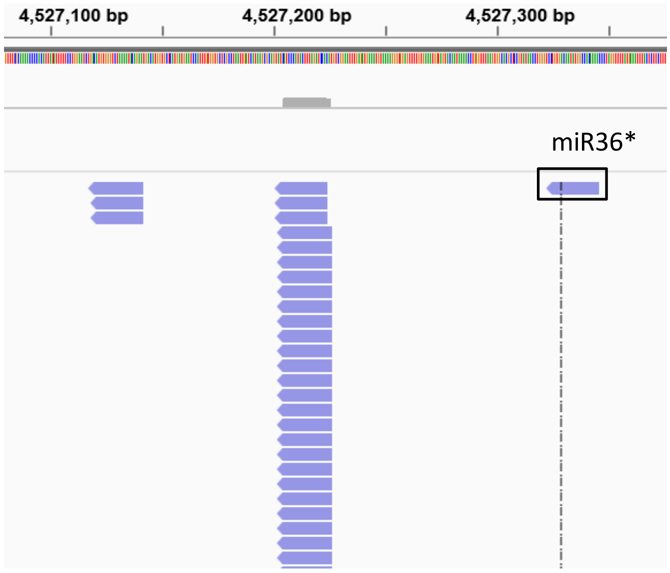

miR36 (89%)

miR36 precursor

scaffold	Position
5	4,527,059-4,527,292(-)

>nov-sit-miR44

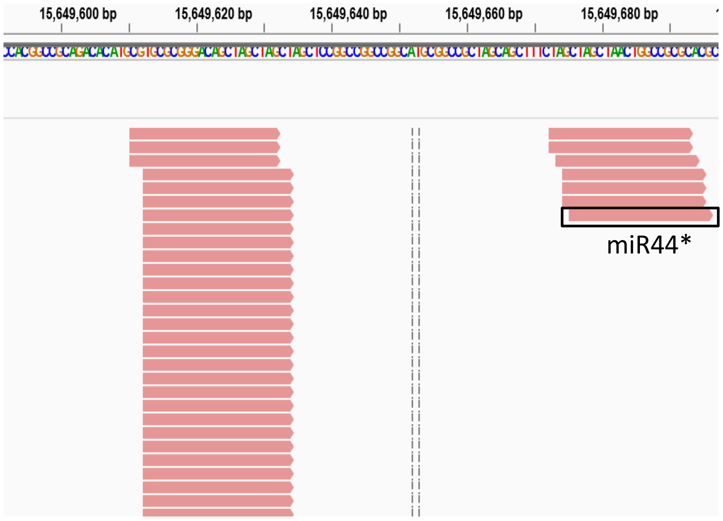

miR44 (86%)

miR44 precursor

scaffold	Position
4	15,649,609-15,649,696(+)
