# Supplementary material for: Genome-wide characterization of microRNA in foxtail millet (Setaria italica)
Source: BMC Plant Biol. 2013 Dec 13;13:212. doi: 10.1186/1471-2229-13-212 (PMC3878754; doi:10.1186/1471-2229-13-212)
Supplement: Additional file 11 — Primers used in this study. [file 1471-2229-13-212-S11.pdf]

Additional file 11: Primers used in this study

Stem-loop RT-PCR Primers (The nov-sit-miRNAs validated by stem-loop RT-PCR)

nov-sit-miR103	miRNA sequence	5'-GCTCACTTCTCTGTCTGTCAGC-3'
	RT primer	5'-GTCGTATCCAGTGCAGGGTCCGAGGTATTCGCACTGGATACGACGCTGACAG-3'
	Forward primer	5'-CCCGTGGGCTCACTTCTCTGT-3'
nov-sit-miR105	miRNA sequence	5'-GCTCACTCCTCTTTCTGTCAGC-3'
	RT primer	5'-GTCGTATCCAGTGCAGGGTCCGAGGTATTCGCACTGGATACGACGCTGACAG-3'
	Forward primer	5'-TGGCGG GCTCACTCCTCTTT-3'
nov-sit-miR21	miRNA sequence	5'-TTCGGACCAGGCTTCATTCCCC-3'
	RT primer	5'-GTCGTATCCAGTGCAGGGTCCGAGGTATTCGCACTGGATACGACGGGGAATG-3'
	Forward primer	5'-GCGGCTTTCGGACCAGGCTT-3'
nov-sit-miR50	miRNA sequence	5'-TGAGCCGAACCAATATCACTC-3'
	RT primer	5'-GTCGTATCCAGTGCAGGGTCCGAGGTATTCGCACTGGATACGACGAGTGATA-3'
	Forward primer	5'-GCACCTCTGAGCCGAACCAAT-3'
nov-sit-miR58	miRNA sequence	5'-TCGGACCAGGCTTCATTCCCCT-3'
	RT primer	5'-GTCGTATCCAGTGCAGGGTCCGAGGTATTCGCACTGGATACGACAGGGGA-3'
	Forward primer	5'-GCCGTTCGGACCAGGCTTCAT-3'
nov-sit-miR64	miRNA sequence	5'-TCCACAGGCTTTCTTGAACGA-3'
	RT primer	5'-GTCGTATCCAGTGCAGGGTCCGAGGTATTCGCACTGGATACGACTCGTTC-3'
	Forward primer	5'-GCGGCGTCCACAGGCTTTCTT-3'
nov-sit-miR31	miRNA sequence	5'-TTAGGCTCGGGGACTATGGTG-3'
	RT primer	5'-GTCGTATCCAGTGCAGGGTCCGAGGTATTCGCACTGGATACGACCACCAT-3'
	Forward primer	5'-ACGCGTTTAGGCTCGGGGACT-3'
nov-sit-miR14	miRNA sequence	5'-TTGAGCCGCGTCAATATCTCC-3'
	RT primer	5'-GTCGTATCCAGTGCAGGGTCCGAGGTATTCGCACTGGATACGACGGAGAT-3'
	Forward primer	5'-CCTACCTTGAGCCGCGTCAAT-3'
Universal	Reverse primer	5'-GTGCAGGGTCCGAGGT-3'

Stem-loop RT-PCR Primers (The miRNAs (whose targets have validated) validated by stem-loop RT-PCR)

sit-miR160	miRNA sequence	5'-TGCCTGGCTCCCTGTATGCCA-3'
	RT primer	5'-GTCGTATCCAGTGCAGGGTCCGAGGTATTCGCACTGGATACGACTGGCAT-3'
	Forward primer	5'-CCCGTGGTGCCTGGCTCCCTGT-3'
sit-miR171a	miRNA sequence	5'-TGATTGAGCCGCGCCAATATC-3'
	RT primer	5'-GTCGTATCCAGTGCAGGGTCCGAGGTATTCGCACTGGATACGACGATATT-3'
	Forward primer	5'-GAATGTGATTGAGCCGCGCC-3'
nov-sit-miR14	miRNA sequence	5'-TTGAGCCGCGTCAATATCTCC-3'
	RT primer	5'-GTCGTATCCAGTGCAGGGTCCGAGGTATTCGCACTGGATACGACGGAGAT-3'
	Forward primer	5'-CCTACCTTGAGCCGCGTCAAT-3'
nov-sit-miR15	miRNA sequence	5'-TTGAGCCGCGCCAATATCTCT-3'
	RT primer	5'-GTCGTATCCAGTGCAGGGTCCGAGGTATTCGCACTGGATACGACAGAGATAT-3'
	Forward primer	5'-TATTTTTTGAGCCGCGCCA-3'
nov-sit-miR49	miRNA sequence	5'-TGAGCCGAGCCAATATCACT-3'
	RT primer	5'-GTCGTATCCAGTGCAGGGTCCGAGGTATTCGCACTGGATACGACAGTGAT-3'

	Forward primer	5'-CCGCTGTGAGCCGAGCCAAT-3'
Universal	Reverse primer	5'-GTGCAGGGTCCGAGGT-3'

Stem-loop RT-PCR Primers (Stem-loop RT-PCR validations of the smRNA-seq results)

sit-miR156a	miRNA sequence	5'-TGACAGAAGAGAGTGAGCACA-3'
	RT primer	5'-GTCGTATCCAGTGCAGGGTCCGAGGTATTCGCACTGGATACGACTGTGCT-3'
	Forward primer	5'-CCCGTGGTGACAGAAGAGAGTG-3'
sit-miR156c	miRNA sequence	5'-GCTCGCTCCTCTTTCTGTCAGC-3'
	RT primer	5'-GTCGTATCCAGTGCAGGGTCCGAGGTATTCGCACTGGATACGACGCTGAC-3'
	Forward primer	5'-CCCGTGGGCTCGCTCCTCTTTCT-3'
sit-miR164c	miRNA sequence	5'-TGGAGAAGCAGGACACGTGAG-3'
	RT primer	5'-GTCGTATCCAGTGCAGGGTCCGAGGTATTCGCACTGGATACGACCTCACG-3'
	Forward primer	5'-CCGTGGTGGAGAAGCAGGACA-3'
sit-miR166b	miRNA sequence	5'-TCTCGGACCAGGCTTCATTCC-3'
	RT primer	5'-GTCGTATCCAGTGCAGGGTCCGAGGTATTCGCACTGGATACGACGGAATG-3'
	Forward primer	5'-CGTTATCTCTCGGACCAGGCTT-3'
sit-miR166d	miRNA sequence	5'-TCGGACCAGGCTTCAATCCCT-3'
	RT primer	5'-GTCGTATCCAGTGCAGGGTCCGAGGTATTCGCACTGGATACGACAGGGAT-3'
	Forward primer	5'-CGTTATCTCGGACCAGGCTTCA-3'
nov-sit-mir31	miRNA sequence	5'-TTAGGCTCGGGGACTATGGTG-3'
	RT primer	5'-GTCGTATCCAGTGCAGGGTCCGAGGTATTCGCACTGGATACGACCACCAT-3'
	Forward primer	5'-AAGGTGGTTAGGCTCGGGGACT-3'
nov-sit-miR64	miRNA sequence	5'-TCCACAGGCTTTCTTGAACGA-3'
	RT primer	5'-GTCGTATCCAGTGCAGGGTCCGAGGTATTCGCACTGGATACGACTCGTTC-3'
	Forward primer	5'-GCGGCGTCCACAGGCTTTCTT-3'
nov-sit-miR103	miRNA sequence	5'-GCTCACTTCTCTGTCTGTCAGC-3'
	RT primer	5'-GTCGTATCCAGTGCAGGGTCCGAGGTATTCGCACTGGATACGACGCTGACAG-3'
	Forward primer	5'-CCCGTGGGCTCACTTCTCTGT-3'
nov-sit-mir141	miRNA sequence	5'-ATGATTGCAAGTCTTGCAGCA-3'
	RT primer	5'-GTCGTATCCAGTGCAGGGTCCGAGGTATTCGCACTGGATACGACTGCTGC-3'
	Forward primer	5'-CCCGTGGATGATTGCAAGTCTT-3'
nov-sit-mir149	miRNA sequence	5'-AGTGAATGAAGCGGGAGGTAA-3'
	RT primer	5'-GTCGTATCCAGTGCAGGGTCCGAGGTATTCGCACTGGATACGACTTACCT-3'
	Forward primer	5'-ATAGGTGGAGTGAATGAAGCGGG-3'
U6	RT primer	5'-GTGCAGGGTCCGAGGTTTTGGACCATTTCTCGAT-3'
	Forward primer	5'-GGAACGATACAGAGAAGATTAGCA-3'
Universal	Reverse primer	5'-GTGCAGGGTCCGAGGT-3'

5’RACE Primers

Si016508m	GeneRacer 5' primer:	5'-CGACTGGAGCACGAGGACACTGA-3'
	Reverse GSP primer	5'-CCAGTGGATGGTGCGAAGCAGTTGAT-3'
	GeneRacer 5' Nested primer	5'-GGACACTGACATGGACTGAAGGAGTA-3'
	Reverse Nested GSP primer	5'-CAAAGGCAGTGAGCTTAACGAACGGCAGA-3'
Si005991m	GeneRacer 5' primer:	5'-CGACTGGAGCACGAGGACACTGA-3'

	Reverse GSP primer	5'-CGCTGCTTGCCGTGTCCGTTAGTATG-3'
	GeneRacer 5' Nested primer	5'-GGACACTGACATGGACTGAAGGAGTA-3'
	Reverse Nested GSP primer	5'-CGGGAAGCCCCCACCAGTACCTAATGAT-3'
Si034525m	GeneRacer 5' primer:	5'-CGACTGGAGCACGAGGACACTGA-3'
	Reverse GSP primer	5'-CCGTTTCCAGTAACCTCAGGGGAGAATGT-3'
	GeneRacer 5' Nested primer	5'-GGACACTGACATGGACTGAAGGAGTA-3'
	Reverse Nested GSP primer	5'-CGATTGCGGAGTGATAGCATCAAGTCGG-3'
Si016509m	GeneRacer 5' primer:	5'-CGACTGGAGCACGAGGACACTGA-3'
	Reverse GSP primer	5'-CGTGCCGACTTGACGAAAACGCTGAAC-3'
	GeneRacer 5' Nested primer	5'-GGACACTGACATGGACTGAAGGAGTA-3'
	Reverse Nested GSP primer	5'-CTCATCTGCTGCTCGGTCAGTATGGTCC-3'
